# Rhythm and ROS: Hepatic Chronotherapeutic Features of Grape Seed Proanthocyanidin Extract Treatment in Cafeteria Diet-Fed Rats

**DOI:** 10.3390/antiox12081606

**Published:** 2023-08-12

**Authors:** Antonio J. Cortés-Espinar, Néstor Ibarz-Blanch, Jorge R. Soliz-Rueda, Béatrice Bonafos, Christine Feillet-Coudray, François Casas, Francisca Isabel Bravo, Enrique Calvo, Javier Ávila-Román, Miquel Mulero

**Affiliations:** 1 Nutrigenomics Research Group, Department of Biochemistry and Biotechnology, Universitat Rovira i Virgili, 43007 Tarragona, Spain; antoniojesus.cortes@estudiants.urv.cat (A.J.C.-E.); nestor.ibarz@urv.cat (N.I.-B.); jorgericardo.soliz@fundacio.urv.cat (J.R.S.-R.); franciscaisabel.bravo@urv.cat (F.I.B.); enrique.calvo@urv.cat (E.C.); 2 Nutrigenomics Research Group, Institut d’Investigació Sanitària Pere Virgili, 43007 Tarragona, Spain; 3 DMEM, EMN, UMR 866, INRAe, Université de Montpellier, 34090 Montpellier, France; beatrice.bonafos@inrae.fr (B.B.); christine.coudray@inrae.fr (C.F.-C.); francois.casas@inrae.fr (F.C.); 4 Molecular and Applied Pharmacology Group (FARMOLAP), Department of Pharmacology, Universidad de Sevilla, 41012 Sevilla, Spain

**Keywords:** oxidative stress, phenolic compounds, proanthocyanidins, GSPE, liver, cafeteria diet, diurnal rhythms, circadian rhythms, zeitgeber, chronotherapy

## Abstract

Polyphenols play a key role in the modulation of circadian rhythms, while the cafeteria diet (CAF) is able to perturb the hepatic biological rhythm and induce important ROS production. Consequently, we aimed to elucidate whether grape seed proanthocyanidin extract (GSPE) administration recovers the CAF-induced hepatic antioxidant (AOX) misalignment and characterize the chronotherapeutic properties of GSPE. For this purpose, Fischer 344 rats were fed a standard diet (STD) or a CAF and concomitantly treated with GSPE at two time-points (ZT0 vs. ZT12). Animals were euthanized every 6 h and the diurnal rhythms of hepatic ROS-related biomarkers, hepatic metabolites, and AOX gene expression were examined. Interestingly, GSPE treatment was able to recover the diurnal rhythm lost due to the CAF. Moreover, GSPE treatment also increased the acrophase of *Sod1*, as well as bringing the peak closer to that of the STD group. GSPE also corrected some hepatic metabolites altered by the CAF. Importantly, the differences observed at ZT0 vs. ZT12 due to the time of GSPE administration highlight a chronotherapeutic profile on the proanthocyanin effect. Finally, GSPE could also reduce diet-induced hepatic oxidative stress not only by its ROS-scavenging properties but also by retraining the circadian rhythm of AOX enzymes.

## 1. Introduction

Biological rhythms are behavioral, physiological, and molecular functions that are synchronized with the external environment. The most studied biological rhythm is the circadian rhythm, which allows organisms to adapt to changes caused by the light/day cycle in a 24-h period. That rhythm arose in response to the Earth’s rotation movement, which dictates daily variations in light and temperature [1,2]. As a result, diurnal oscillations have been reported in different processes such as sleep–wake cycles, hormone production, metabolism, and immune function, among others. Furthermore, these periodical variations are markedly conserved throughout evolution [3]. Therefore, one of the fundamental characteristics of circadian rhythms is their adaptability to changes in the environment. In fact, circadian rhythms are synchronized by external cues, also known as *zeitgebers* (ZT), such as the presence or absence of light, food intake, or exercise, among others [3,4]. Light, which is the most important ZT, is recognized by cells found in the retina, and a transduction signal is sent to the suprachiasmatic nucleus (SCN) of the hypothalamus, resetting the central clock in mammals. Additionally, the SCN is also responsible for synchronizing all the peripheral clocks present in organs such as the liver, skeletal muscle, and adipose tissue, thereby acting as the master regulator of circadian rhythms [5].

The molecular mechanism behind the circadian clock involves a network of transcriptional-translational feedback loops (TTFL), mainly controlled by the gene’s circadian locomotor output cycles kaput (*Clock*) and aryl hydrocarbon receptor nuclear translocator-like protein1 (*Arntl1*, but usually called *Bmal1*), which act as positive regulators; and period (*Per*) and cryptochrome (*Cry*), which act as negative regulators [4,6]. Consequently, the TTFL regulates the expression of several clock-controlled genes (CCGs) promoting a circadian rhythmic expression. However, many authors have shown that unhealthy lifestyle habits could promote altered rhythmic expression of the TTFL genes. For example, Salgado-Delgado et al. showed that rats that ate during the resting phase reversed the rhythm of *Clock*, *Bmal1*, and *Per1* in the liver [7]. Additionally, Kohsaka et al. also demonstrated that a high-fat diet altered the expression of the clock genes *Clock*, *Bmal1*, and *Per2* in adipose tissue and liver, but not in the hypothalamus; promoting desynchronization between the central and the peripheral clocks [8]. Since a mutual relationship between the circadian machinery and metabolism has been established, these rhythm desynchronizations could influence the promotion of metabolic pathological conditions such as obesity or metabolic syndrome (MetS) [9].

In this respect, MetS, which is a cluster of physiological, biochemical, and metabolic factors, can increase the risk of developing other pathologies, such as cardiovascular disease, non-alcoholic fatty liver disease (NAFLD), or type II diabetes [10,11,12]. In fact, MetS is characterized by several factors, such as insulin resistance, hypertension, hyperlipidemia, and abdominal obesity. MetS and obesity are also associated with low-grade chronic inflammation and oxidative stress, which are key mechanisms in the development of their associated pathologies. In this sense, obesity induces an excess of adipose tissue (hypertrophy and hyperplasia), which triggers the release of pro-inflammatory cytokines that can recruit immune cells and promote an increase in reactive oxygen species (ROS) and inflammation [13,14]. The accumulation of ROS leads to oxidative stress, which promotes damage to biomolecules, tissues, and organs. Consequently, organisms have developed AOX defenses to combat the damage caused by free radicals, in particular (a) the enzymatic AOX defenses, such as superoxide dismutases (SODs), catalase, glutathione peroxidase (GPx1), or glutathione-disulfide reductase (GSR); and (b) the AOX chemical defenses, such as vitamin E or glutathione (GSH), which behaves as an electron donor [15]. Interestingly, certain free-radical defense mechanisms have also been shown to be influenced by circadian rhythms through the regulation of AOX gene expression; for example, *Sod1* expression is under the control of BMAL1 [16]. Moreover, it has also been shown that the clock machinery protein BMAL1 and NRF2 cooperatively control the oxidative response and redox homeostasis [17].

In this context, there are multiple nutritional approaches to counteract ROS-induced oxidative damage, with polyphenols being one of the main chemical groups responsible for this beneficial dietetic effect [18,19,20,21]. More concisely, phenolic compounds are secondary metabolites produced by plants under changes in environmental conditions (e.g., drought, temperature, heavy metals, salinity, and ultraviolet radiation) or biotic stresses (e.g., aggression by pathogens or herbivores). They are found in a wide variety of plant foods, such as fruits and vegetables, or even beverages such as tea or wine [22]. Their importance relies on their ability to modulate and prevent inflammatory or cardiovascular diseases, particularly through their effects on the regulation of free radicals [23]. In this context, it has been demonstrated that supplementation with grape seed proanthocyanidin extract (GSPE), a mixture of flavonoids that includes oligomeric proanthocyanidins obtained from grape seeds and skin, can act as an anti-inflammatory agent, antimicrobial, or even as an appetite suppressant [24,25]. Furthermore, many authors have shown that GSPE can also affect metabolism by improving insulin resistance or lipid metabolism, among others [26,27]. Additionally, GSPE exhibits potent AOX activity, effectively reducing cellular levels of free radicals [28,29]. Moreover, recent studies have shown that GSPE can also influence the circadian machinery. In reference to this, previous studies of our group have shown that GSPE was able to modulate the expression pattern of *Bmal1* and *Nampt* genes in the hypothalamus and the liver of rats, thus influencing gene expression in the central pacemaker and liver peripheral clock [30,31].

On the other hand, it has been suggested that the timing of GSPE intake may also influence its beneficial effects through different mechanisms (e.g., microbiota). This has also been shown for other polyphenols; for example, the timing of resveratrol consumption may be important to provide positive health effects. A mouse study on resveratrol measured AOX effects based on the time of day of dosing. Resveratrol administered during the active period (dark for mice) acted as a strong AOX in a dose-dependent manner, with doses ranging from 0.8 to 5 mg/kg, which converts into a physiological dose for humans. In contrast, when resveratrol was administered during the inactive period (light for mice), it became a pro-oxidant, increasing oxidative stress in a dose-dependent manner [32].

In consequence, in the present study, Fischer 344 rats were used to investigate the following objectives: (a) To evaluate whether the cafeteria diet (CAF) is able to disturb the hepatic diurnal rhythmicity of AOX-related parameters; (b) to elucidate whether the dietary oral administration of GSPE is capable of recovering the hypothetical CAF-induced AOX rhythm misalignment in the liver; and finally (c) to characterize the chronotherapeutic properties of GSPE (differential effects depending on the time of administration).

## 2. Materials and Methods

### 2.1. Grape Seed Proanthocyanidin Extract (GSPE)

GSPE was provided by Les Dérives Résiniques et Terpéniques (Dax, France) and was obtained from white grape seeds and skins. Our group has previously described the phenolic composition of the extract used for this study [33], which was mainly composed of catechin, epicatechin, dimers, trimers, and tetramers of procyanidins or epicatechin gallate, among others.

### 2.2. Animals and Experimental Procedure

**Figure 1:**
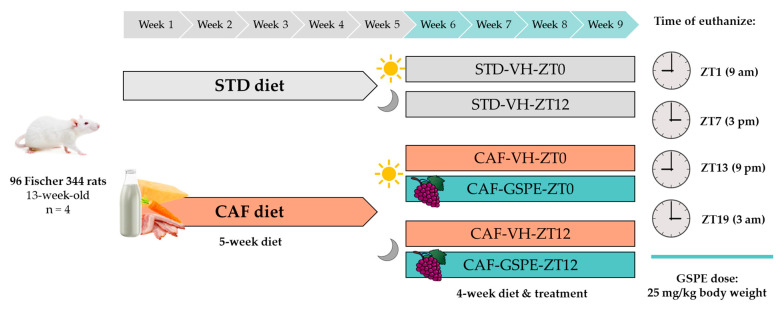
Experimental design. Ninety-six Fischer 344 rats were randomly divided into two groups based on their diet: 32 were fed STD and 64 were fed CAF for 5 weeks. Then, each diet group was further divided into two groups for STD-fed rats and four groups for CAF-fed rats, to receive either VH or GSPE doses. Both STD and CAF-fed rats were administered VH at two different time points: 8 a.m. (ZT0) and 8 p.m. (ZT12), while CAF-fed rats were additionally given GSPE (25 mg/kg b.w.) at the same time points. Finally, each experimental group was subdivided into four based on the time of death of the animals: 9 a.m. (ZT1), 3 p.m. (ZT7), 9 p.m. (ZT13), and 3 a.m. (ZT19).

Ninety-six 12-week-old male Fischer 344 rats (Janvier, Barcelona, Spain) were housed in pairs under standard conditions (23 °C, 55% humidity, and a standard photoperiod, 12 h light–12 h dark) with ad libitum access to food and drink water. After a week of the acclimatization period, animals were weighed and randomly divided into two groups according to the diet: 32 rats were fed an STD (kcal/100 g: 20% protein, 8% fat, and 72% carbohydrates, Safe-A04c, Scientific Animal Food and Engineering, Barcelona, Spain) and 64 rats were fed a CAF (kcal/100 g: 11% protein, 31% fat, and 58% carbohydrates) during a 5-week pre-treatment. The CAF consisted of food typically consumed by humans, with poor nutritional value but high palatability and high caloric content, including biscuits with cheese and pâté, bacon, *ensaimada* (pastry), standard chow, carrots, and milk containing 22% sucrose (*w*/*v*). This diet has been extensively used in the literature to induce obesity and MetS [34,35,36]. Treatment started in the fifth week and lasted 4 weeks until animals were euthanized. Throughout the experiment, body weight (b.w.) and food intake were recorded weekly. Finally, rats were fasted for 3 h and then anesthetized with 3% isoflurane and euthanized by decapitation. Each experimental group was divided into four sub-groups of four rats according to the time of death of the animals (9 a.m. (ZT1), 3 p.m. (ZT7), 9 p.m. (ZT13), or 3 a.m. (ZT19)). Figure 1 provides a detailed scheme of the study design. Blood was collected and serum was obtained after centrifugation (2000× *g* and 4 °C, 15 min). The serum and liver were kept at −80 °C for further studies. All animal care and experimental protocols were approved by the Ethics Review Committee for Animal Experimentation of the Universitat Rovira i Virgili (reference number 9495, 18 September 2019) and were conducted in accordance with Directive 86/609EEC of the Council of the European Union and the procedure established by the Departament d’Agricultura, Ramaderia i Pesca of the Generalitat de Catalunya.

### 2.3. Dosage Information/Dosage Regimen

During the last 4 weeks of the experiment, rats received a daily oral dose of either VH (condensed milk diluted in water in a ratio of 1:5 *v*/*v*) or GSPE diluted in VH (25 mg/kg b.w.). This dose of GSPE has been shown to be the lowest and most efficient that modulates most central metabolic pathways in healthy rats, being regularly employed by our group [37]. Furthermore, the dose of GSPE at 25 mg/kg per day is equivalent to an intake of approximately 370 mg of phenols per day, which can be easily achieved by polyphenol-rich diets (such as the Mediterranean) after considering the conversion of animal (rat) to human [38]. VH for STD-fed rats and VH or GSPE for CAF-fed rats were administered either at ZT0 (8 a.m.) or at ZT12 (8 p.m.). The rats were euthanized 3 h after the last dose by decapitation after 3% isoflurane inhalation.

### 2.4. Hepatic RNA Extraction

RNA extraction was carried out from a 20-30 mg portion from the previously isolated hepatic tissue, which was mixed with the Trizol^®^ reagent (Thermo Fisher, Madrid, Spain) and homogenized by Tissue Lyser LT (Qiagen, Madrid, Spain). The homogenate supernatant was placed into a new Eppendorf tube after a 10 min centrifugation (12,000× *g* at 4 °C). Then, 250 μL of chloroform was added, and after a 15 min centrifugation (12,000× *g* at 4 °C), the aqueous phase was placed into a new Eppendorf tube. Later, 500 μL of isopropanol was added, and a 10 min centrifugation was performed. The pellet was washed twice with 70% ethanol and centrifuged for 5 min (8000I× *g* at 4 °C). Dry washed pellet was resuspended in 60 μL of nuclease-free water. Finally, RNA yield and purity were assessed on a NanoDrop 1000 spectrophotometer (Thermo Scientific, Wilmington, DE, USA). 

### 2.5. cDNA Synthesis and Gene-Expression Analysis

In order to analyze sample gene expression, cDNA was synthesized from RNA using the High-Capacity cDNA Reverse Transcription Kit (Applied Biosystems, Barcelona, Spain). The reaction was performed according to the instructions of the manufacturer in a Galaxy XP ClearLine Thermal Cycler (ClearLine, Dominique Dutscher, Brumath France). The cDNA was subjected to a quantitative polymerase chain reaction amplification using iTaq Universal SYBR Green Supermix (Bio-Rad, Madrid, Spain) in a CFX96 Touch Real-Time PCR Detection System (Bio-Rad, Madrid Spain). The nucleotide sequence of primers used for the different genes is described in Table 1 and was obtained from Biomers.net (Ulm, Germany). mRNA relative expression was represented as a fold change that was calculated as a percentage of the ZT0-STD-VH at ZT1 group using the 2^−∆∆^Ct method with Peptidylprolyl Isomerase A (*PPIA*) gene as endogenous control, as reported by Schmittgen and Livak [39].

### 2.6. Serum Biochemical Analysis

Enzymatic colorimetric assays were used for the analysis of some biochemical parameters on serum samples such as glucose, total cholesterol (TC), and triglycerides (TAG) (QCA, Amposta, Tarragona, Spain) and non-esterified free fatty acids (NEFAs) (WAKO, Neuss, Germany), according to the manufacturer’s instructions.

### 2.7. Determination of AOX Activities and Lipid and Protein Oxidation in Liver Tissue

Liver samples were homogenized in cold phosphate buffer (50 mM, pH 7) (0.5 g liver tissue per 4.5 mL phosphate buffer) by using an Ultra-Turrax T25 homogenizer (IKA, Staufen, Germany). Then, the homogenate was centrifuged for 10 min at 1000× *g* at 4 °C, and supernatants were collected for liver enzymatic analysis. Total superoxide dismutase (SOD) was measured according to the method of Marklund [40]. Catalase activity was measured according to the method of Beers and Sizer [41]. Glutathione peroxidase (GPx1) was measured according to the method of Flohe and Gunzler [42]. Total glutathione (GSH) was measured according to the method of Griffith [43]. Protein oxidation (thiols) was assessed by the measurement of sulfhydryl groups [44] and advanced oxidation protein products [45].

### 2.8. Metabolomics Analysis

The metabolomic analysis of rat liver samples was performed at the Centre for Omic Sciences (COS, Tarragona, Spain) by using gas chromatography coupled with quadrupole time-of-flight mass spectrometry (GC-qTOF model 7200, Agilent, Santa Clara, CA, USA). Sample extraction was performed by adding 400 μL of methanol:water in an 8:2 proportion containing the internal standard mixture to liver samples (approximately 10–20 mg). Later, samples were mixed and homogenized using a stainless-steel ball on a bullet blender, incubated for 10 min at 4 °C, and centrifuged at 19,000× *g*. Supernatants were evaporated to dryness before compound derivatization (methoximation and silylation). GC-qTOF was used for analyzing derivatized compounds. The Fiehn Method [46] was used to carry out the chromatographic separation, using a J&W Scientific HP5-MS film capillary column (30 m × 0.25 mm × 0.25 μm, Agilent, Santa Clara, CA, USA) and helium as carrier gas with an oven program from 60 to 325 °C. Ionization was conducted by electronic impact (EI), operating in full-scan mode and electron energy of 70 eV. Metabolite identification was performed using commercial standards and by matching their EI mass spectrum and retention time to a metabolomic Fiehn library (from Agilent, Santa Clara, CA, USA), in which more than 1400 metabolites are included. After putative identification of metabolites, they were semi-quantified in terms of the internal standard response ratio.

### 2.9. Statistical Analysis

Data are expressed as Mean ± Standard Deviation (SD) or Median and Interquartile Range (IQR). In all cases, the Shapiro–Wilk test was used to verify the normality of the data. The statistical test used for individual analyses is provided in the figure legends. Graphics were performed using GraphPad Prism v. 8 software (GraphPad Software, San Diego, CA, USA) and statistical analysis using SPSS version 26 (IBM Inc, Armonk, NY, USA). For all analyses, a probability value of (*p*) < 0.05 was considered statistically significant.

The influence of diet and treatments, as well as the treatment administration time on the liver metabolomics profile, was assessed through heatmaps analysis using MetaboAnalyst v.5.0 (McGill University, Montreal, QC, Canada) [47].

To analyze the circadian rhythms, we used the Cosinor-based rhythmometry method. To do this, J.R. S-R created a script using PyCharm software (v.2018.2.4, JetBrains s.r.o., Prague, Czech Republic) with Python version 3.7.4. The circadian rhythm was estimated using the CosinorPy package (v.1.1) [48]. To determine the presence of a diurnal rhythm, we considered each gene expression model that fits cosine curves with a significance level of *p* < 0.05. Additionally, this method enabled us to obtain rhythmicity parameters such as the MESOR (average value adjusted for diurnal rhythm), amplitude (the difference between a wave’s peak and average), or acrophase (the time when the oscillation peak occurs) of the diurnal oscillations.

## 3. Results

### 3.1. GSPE Administration at ZT12 Reduces Body Weight Gain in CAF-Fed Animals and Partially Ameliorates Serum Parameters

**Figure 2:**
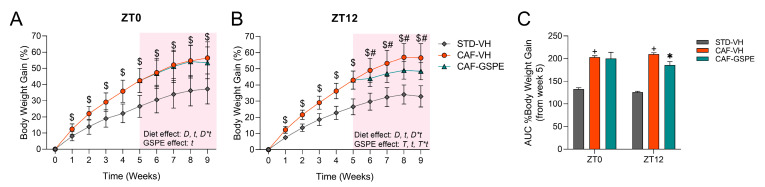
Body weight gain (% to Week 0). (**A**) Body weight gain was expressed in the percentage of ZT0-treated rats (STD-VH, CAF-VH, and CAF-GSPE) during the nine weeks of the experiment. (**B**) Body weight gain was expressed in the percentage of ZT12-treated rats (STD-VH, CAF-VH, and CAF-GSPE) during the nine weeks of the experiment. Data are shown as mean ± S.D (*n* = 14–16). $ indicates significant differences by Student’s *t*-test between STD-VH vs. CAF-VH (*p* < 0.001). # indicates significant differences by Student’s *t*-test between CAF-VH vs. CAF-GSPE (*p* < 0.001). *t*, indicates time effect; *D*, diet effect, D*t, interaction between diet and time, *T*, indicates treatment effect, and T*t, interaction between treatment and time via 2-way ANOVA. (**C**) AUC of the percentage of the BWG from week 5. Data are shown as mean ± S.E.M (*n* = 14–16). + indicates significant differences by Student’s *t*-test between STD-VH vs. CAF-VH (*p* < 0.05). * Indicates significant differences by Student’s *t*-test between CAF-VH vs. CAF-GSPE (*p* < 0.05). STD, rats fed a Standard diet; CAF, rats fed a Cafeteria diet; VH, rats administered vehicle; GSPE, rats administered 25 mg/kg b.w. grape seed proanthocyanidin extract.

Initially, we checked the obesogenic effect of the CAF by measuring the body-weight gain (BWG) weekly, as well as the food intake. In this regard, rats fed a CAF showed a significant increase in BWG and food intake compared to rats fed an STD (Figure 2). In this case, the BWG increased during the first five weeks of the experiment in CAF-fed rats when compared to the STD-fed groups. In addition, food intake was increased in the CAF-fed rats during this time by more than 2.5-fold compared to the STD group (Table S1). Daily caloric consumption also increased during the experimental five-week more than 4-fold in CAF rats vs. STD rats. Then, in the fifth week, rats were treated with VH or GSPE at two separate times: ZT0 (8 a.m.) and ZT12 (8 p.m.). Regarding the CAF-VH group, animals maintained their increase in the BWG and food intake when compared to the STD-VH group throughout the last four weeks of the experiment, regardless of the time of VH administration. Furthermore, the assessment of the Area Under the Curve (AUC) showed an increase in the BWG, which corroborated the obesogenic effect of CAF (Figure 2C). Subsequently, we investigated whether GSPE could mitigate some of the negative effects caused by the CAF. Additionally, we examined whether the time of administration of GSPE (morning/evening) offered any differential effect. As presented in Figure 2A, no differences were observed in BWG or food intake between the CAF-VH and CAF-GSPE groups when treated at ZT0 (8 a.m.). However, at Z12, the CAF-GSPE group showed a significant decrease in BWG compared to the CAF-VH group throughout the 4 weeks of treatment when the animals were treated at ZT12 (8 p.m.) (Figure 2B,C). Nevertheless, there were no changes in food intake between the CAF groups when GSPE was administered at ZT12.

Table 2 shows the results of a second set of experiments in which we evaluated the physiological condition of the rats through the analysis of serum biochemical markers such as glucose, cholesterol, triglycerides, and NEFAs. For animals treated at ZT0, the glucose level was significantly increased in the CAF-VH group when compared with the STD-VH group at ZT7 (*p* = 0.028), while at ZT13, this increase was a trend (*p* = 0.057). However, no differences were observed between CAF-GSPE to CAF-VH in any of the studied time points. Regarding triglyceride levels, the CAF-VH group showed a higher level compared to the STD-VH group at ZT1, ZT13, and ZT19 in ZT0-treated rats (*p* = 0.029, *p* = 0.029 and *p* = 0.029, respectively), being these levels almost three times higher in the CAF-VH group. Despite no statistical differences being found, there was a marked reduction in triglyceride levels of CAF-fed rats after the GSPE treatment in all the studied time points. Additionally, neither cholesterol nor NEFAs levels showed differences due to diet or treatment in any of the possible comparisons between time points.

Concerning ZT12-treated rats, the glucose levels of the CAF-VH group were higher at ZT7, ZT13, and ZT19 in comparison to STD-VH (*p* = 0.028, *p* = 0.028, and *p* = 0.028, respectively). GSPE treatment tended to increase the level of glucose in CAF-GSPE compared with CAF-VH at ZT1 (*p* = 0.057). However, GSPE treatment lowered the glucose levels at ZT13 and ZT19 in a non-significant manner compared to CAF-VH. 

Similarly, in ZT0-treated rats, the CAF increased the triglyceride levels (up to a four-fold increase) when comparing CAF-VH vs. STD-VH groups at ZT7, ZT13, and ZT19 (*p* = 0.029, *p* = 0.029 and *p* = 0.029, respectively). However, in this case (ZT0-treated rats), it appears that GSPE treatment lowered triglyceride levels at ZT13 and ZT19. Finally, as previously observed in ZT12-treated rats, the cholesterol and NEFAs levels did not change due to the CAF or GSPE treatment despite the different time points of death.

### 3.2. GSPE Moderately Mitigates the Alterations in the ROS Detoxification System Originated by CAF in the Liver

As mentioned earlier, obesity has been found to elevate baseline levels of oxidative stress, leading to disruptions in the circadian rhythm of various physiological parameters associated with oxidative stress pathways. Consequently, we investigated whether GSPE, beyond its metabolic and AOX properties, was also able to interact with the molecular machinery that controls circadian rhythms, mitigating the negative effects related to ROS caused by the CAF. Firstly, we examined the diurnal rhythmic activity of key enzymes involved in ROS detoxification, such as SOD, Catalase, and GPx1, by using the Cosinor method. Additionally, in order to assess the liver oxidative status, we also analyzed the diurnal rhythmicity of several hepatic oxidative stress markers, such as oxidized proteins (Thiols) level and GSH content. All data are recompiled in Table S2. 

**Figure 3:**
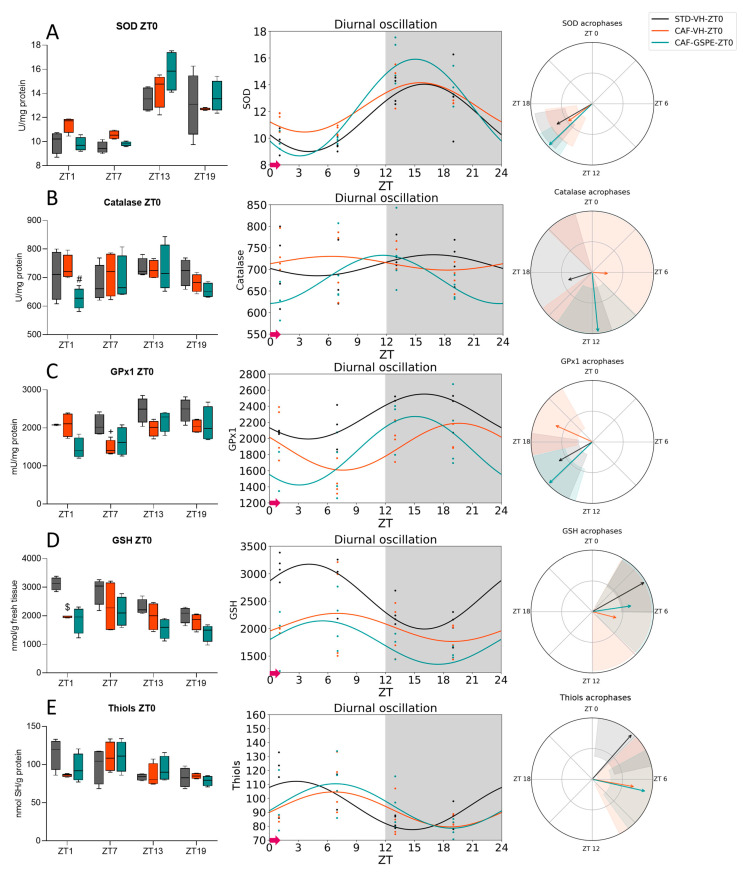
Estimated diurnal rhythms of liver SOD (**A**), Catalase (**B**), GPx1 (**C**), GSH (**D**), and Thiols (**E**) in ZT0-treated rats. Data are shown as the median ± Min to max, its diurnal oscillation, and acrophases with their amplitude. + indicates significant differences (*p* < 0.05) by diet effect (STD-VH vs. CAF-VH), $ indicates tendency (0.1 > *p* ≥ 0.05) by diet effect; # indicates tendency (0.1 > *p* ≥ 0.05) by treatment effect (CAF-VH vs. CAF-GSPE) using Mann–Whitney *U* test. STD, rats fed a Standard diet; CAF, rats fed a Cafeteria diet; VH, rats administered vehicle; GSPE, rats administered 25 mg/kg b.w. grape seed proanthocyanidin extract; Arrow, time of treatment administration.

Regarding ZT0-treated rats, all groups showed diurnal rhythmicity on SOD activity (*p* = 0.01, STD-VH; *p* = 0.002, CAF-VH and *p* < 0.000, CAF-GSPE) peaking at almost the same time, between ZT15 and ZT16 (Figure 3A, Table 3). However, GSPE-treated rats showed a higher acrophase, indicating that the treatment increased the activity of the enzyme. On the other hand, we also observed that while Catalase activity did not show any diurnal rhythm in either STD-VH or CAF-VH (Figure 3B), it tended to present a diurnal rhythm in the CAF-GSPE group (*p* = 0.088). Furthermore, its activity exhibited an amplitude almost 2-fold higher than that reached in the STD-VH group (Table 3), suggesting an enhanced AOX response in the organism. Furthermore, in relation to GPx1 activity (Figure 3C), all three groups (STD-VH, CAF-VH, and CAF-GSPE) showed diurnal rhythmicity (*p* = 0.027, *p* = 0.033, *p* = 0.024, respectively). It is worth highlighting that the STD-VH and CAF-GSPE groups reached their zenith at approximately the same time (ZT15 and ZT16, respectively), while the CAF-VH group did the same at almost ZT20 (Table 3). This suggests that the CAF shifted the acrophase, but GSPE treatment was able to restore the amplitude observed in the STD-VH group. Regarding GSH, the main AOX compound in cells, the STD-VH group showed diurnal rhythmicity (*p* = 0.001) while the CAF-VH group lost this rhythmicity due to the diet (Figure 3D). Nevertheless, CAF-GSPE-treated rats tended to recover the rhythmicity (*p* = 0.063), showing a profile similar to the STD-VH (Table 3). In this regard, only the STD-VH group showed diurnal rhythmicity in thiols (*p* = 0.039). Surprisingly, CAF-GSPE tended to recover the rhythmicity in thiols (*p* = 0.052) but its acrophase shifted for almost 4 h compared to the STD-VH group (Figure 3E).

**Figure 4:**
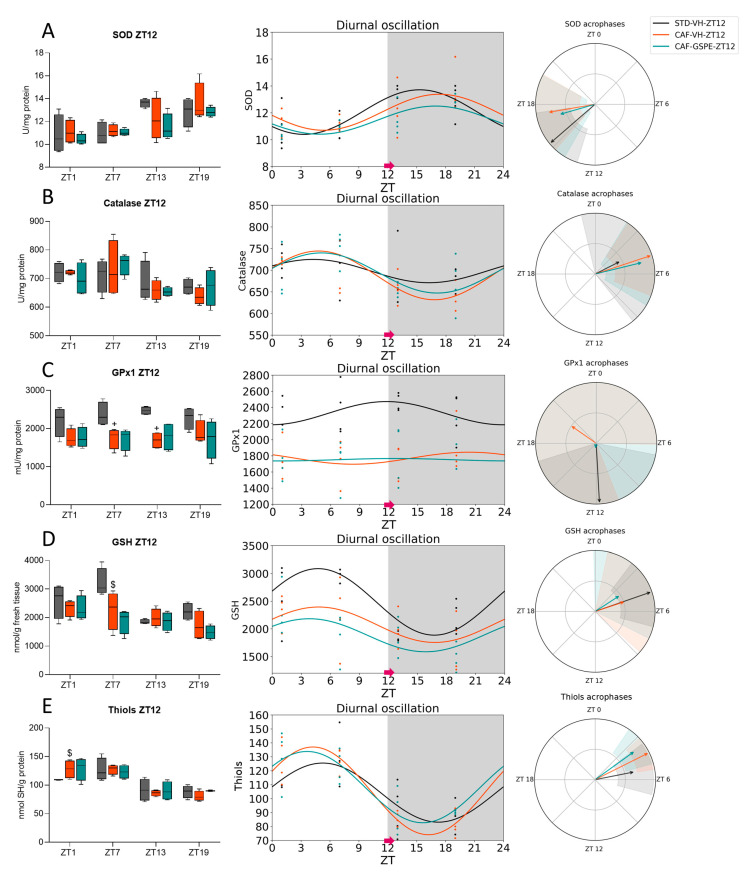
Estimated diurnal rhythms of liver SOD (**A**), Catalase (**B**), GPx1 (**C**), GSH (**D**), and Thiols (**E**) in ZT12-treated rats. Data are shown as the median ± Min to max, its diurnal oscillation, and acrophases with their amplitude. + indicates significant differences (*p* < 0.05) by diet effect (STD-VH vs. CAF-VH), $ indicates tendency (0.1 > *p* ≥ 0.05) by diet effect using Mann–Whitney *U* test. STD, rats fed a Standard diet; CAF, rats fed a Cafeteria diet; VH, rats administered vehicle; GSPE, rats administered 25 mg/kg b.w. grape seed proanthocyanidin extract; Arrow, time of treatment administration.

Regarding ZT12-treated rats, SOD activity showed diurnal rhythmicity in the STD-VH group (*p* = 0.010), while the CAF-VH lost this rhythmicity. Intriguingly, the GSPE treatment was able to restore the diurnal rhythmicity (*p* = 0.014) of its activity (Figure 4A). However, both CAF groups peaked at ZT17, while the STD-fed group did the same 2 h before (Table 4). Furthermore, only the CAF-VH group showed diurnal rhythmicity (*p* = 0.045) on Catalase activity, which could not be recovered by GSPE treatment (Figure 4B). None of the groups showed a diurnal rhythm in the GPx1 activity (Figure 4C), in contrast to what occurred at ZT0. Regarding the oxidative stress marker, GSH (Figure 4D), only the STD-VH group showed a diurnal rhythm, which was disrupted by the CAF, and GSPE treatment was not capable of restoring it. Nevertheless, all groups showed diurnal rhythmicity in thiols levels (*p* = 0.009, STD-VH; *p* < 0.001, CAF-VH and *p* = 0.001, CAF-GSPE), reaching their maximum activity at a similar time-point, between ZT3 and ZT5 (Figure 4E, Table 4).

It is worth highlighting the repetitive pattern observed in all the measured parameters, characterized by the clustering of the AOX-related parameters between the daytime and nighttime phases of the day. Thus, we grouped the data based on the time of death, resulting in two distinct groups: One during the light phase (death time-point ZT1 and ZT7) and the other during the dark period (death time-point ZT13 and ZT19). As shown in Table 5, SOD activity levels were consistently higher during the dark phase compared to the light phase in all groups of rats treated at either ZT0 or ZT12 (*p* = 0.003, STD-VH; *p* = 0.001, CAF-VH and *p* = 0.002, CAF-GSPE for ZT0-treated rats and *p* = 0.008, STD-VH; *p* = 0.021, CAF-VH and *p* = 0.016, CAF-GSPE for ZT12-treated rats). Similarly, the activity of Catalase was also lower during the dark phase vs. the light phase, but only in ZT12-treated rats. In contrast, GPx1 activity exhibited higher activity levels during the dark phase than in the light phase in the ZT0-treated groups (*p* = 0.027, STD-VH; *p* = 0.028, CAF-GSPE).

Regarding oxidative markers, GSH levels were influenced by the daytime phases, showing lower levels during the dark phase than during the light phase, regardless of the time of treatment administration (*p* = 0.002, STD-VH; *p* = 0.037, CAF-GSPE for ZT0-treated rats and *p* = 0.021, STD-VH; *p* = 0.046, CAF-VH for ZT12-treated rats). Furthermore, the level of thiols showed a substantial difference based on the phase of the day, with higher levels during the light phase compared to the dark phase, regardless of the treatment time (*p* = 0.046, STD-VH; *p* = 0.049, CAF-VH; *p* = 0.049, CAF-GSPE for ZT0-treated rats and *p* = 0.005, STD-VH; *p* = 0.001, CAF-VH; *p* = 0.002, CAF-GSPE for ZT12-treated rats). These results suggest that in certain parameters related to ROS, a distinct day/night pattern may also be present.

### 3.3. The ROS Detoxification System in the Liver Is Altered Due to the CAF

**Figure 5:**
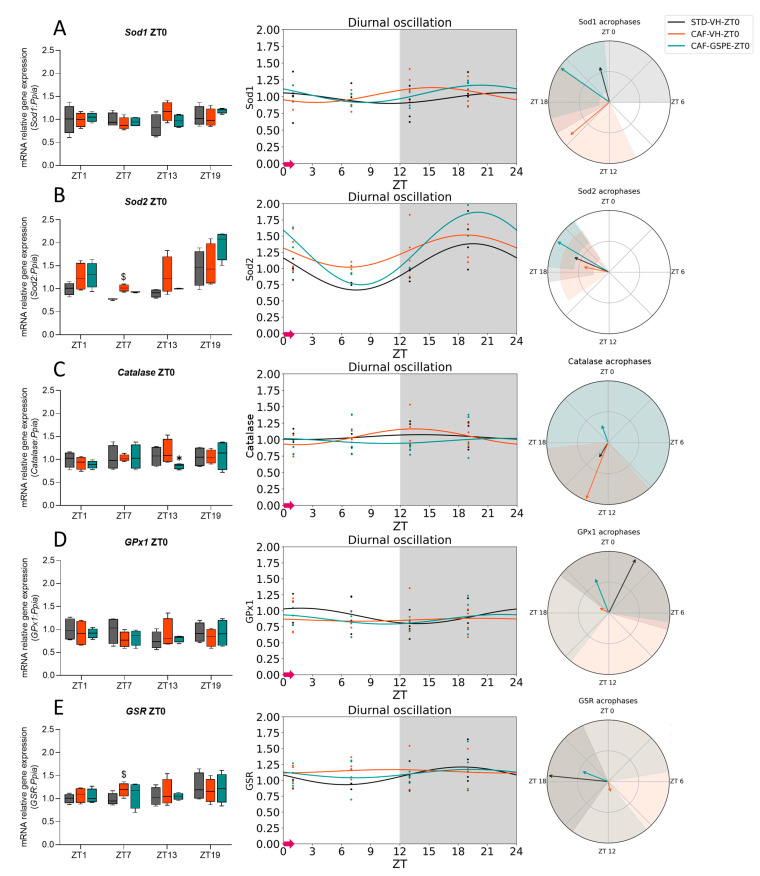
Estimated diurnal rhythms of liver *Sod1* (**A**), *Sod2* (**B**), *Catalase* (**C**), *GPx1* (**D**), and *GSR* (**E**) gene expression in ZT0-treated rats. Data are shown as the median ± Min to max, its diurnal oscillation, and acrophases with their amplitude. $ indicates tendency (0.1 > *p* ≥ 0.05) by diet effect (STD-VH vs. CAF-VH); * indicates significant differences (*p* < 0.05) by treatment effect (CAF-VH vs. CAF-GSPE) using Mann-Whitney *U* test. STD, rats fed a Standard diet; CAF, rats fed a Cafeteria diet; VH, rats administered vehicle; GSPE, rats administered 25 mg/kg b.w. grape seed proanthocyanidin extract; Arrow, time of treatment administration.

**Figure 6:**
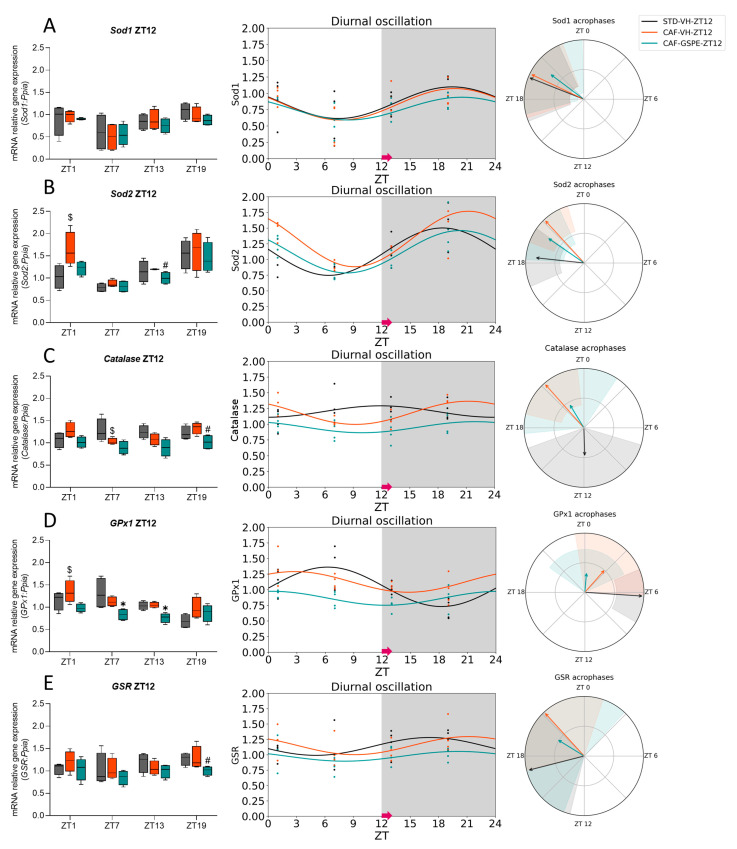
Estimated diurnal rhythms of liver *Sod1* (**A**), *Sod2* (**B**), *Catalase* (**C**), *GPx1* (**D**), and *GSR* (**E**) gene expression in ZT12-treated rats. Data are shown as the median ± Min to max, its diurnal oscillation, and acrophases with their amplitude. $ indicates tendency (0.1 > *p* ≥ 0.05) by diet effect (STD-VH vs. CAF-VH); * indicates significant differences (*p* < 0.05) by treatment effect (CAF-VH vs. CAF-GSPE), # indicates tendency (0.1 > *p* ≥ 0.05) by treatment effect using Mann–Whitney *U* test. STD, rats fed a Standard diet; CAF, rats fed a Cafeteria diet; VH, rats administered vehicle; GSPE, rats administered 25 mg/kg b.w. grape seed proanthocyanidin extract; Arrow, time of treatment administration.

To investigate the rhythmicity of gene expression related to the ROS detoxification system in the liver, we used the Cosinor method to study the expression of several key genes. Specially, we focused on Superoxide dismutase 1 (*Sod1*), mainly found in the cytoplasm, Superoxide dismutase 2 (*Sod2*), predominantly found in the mitochondria, and *Catalase*. Furthermore, the expression of Glutathione peroxidase 1 (*GPx1*) and Glutathione-disulfide reductase (*GSR*), which are essential components of the GSH cycle, were also examined. The data for ZT0-treated rats are represented in Figure 5 and Table 6, while those for ZT12-treated rats are in Figure 6 and Table 7.

Regarding ZT0-treated rats, *Sod1* expression did not exhibit diurnal rhythmicity in either STD-VH or CAF-VH (Figure 5A, Table 6). However, GSPE treatment in the CAF-GSPE group resulted in *Sod1* gene expression that followed a similar pattern to diurnal rhythmicity (*p* = 0.021), thus increasing the response to oxidative stress during the day. Moving on to the *Sod2* expression, the STD-VH group showed rhythmicity (*p* = 0.008), which was lost in the CAF-VH group (Figure 5B). Nonetheless, GSPE treatment effectively restored the diurnal rhythm that was disrupted by CAF (*p* < 0.001) (Table 6). Additionally, both the STD-VH and CAF-GSPE groups peaked at nearly the same time (ZT20), but the zenith was higher in the GSPE-treated group, enhancing the AOX response in the CAF-GSPE group. However, no diurnal rhythmicity was found in the expression of *Catalase* in any group (Figure 5C). Finally, regarding the gene expression of *GPx1* (Figure 5D) and *GSR* (Figure 5E), neither of them showed diurnal rhythmicity in any group.

On the other hand, in the case of ZT12-treated rats, *Sod1* gene expression (Figure 6A) showed or tended to show a diurnal rhythm in all groups (*p* = 0.095 for STD-VH, *p* = 0.047 for CAF-VH, and *p* = 0.083 for the CAF-GSPE group). However, while both STD-VH and CAF-VH peaked at almost ZT19, the CAF-GSPE group showed the acrophase an hour later (ZT20) (Table 7). Additionally, *Sod2* gene expression (Figure 6B) showed diurnal rhythmicity in all groups (*p* = 0.003 for STD-VH, *p* = 0.008 for CAF-VH, and *p* = 0.002 for CAF-GSPE group) but differences were observed in the timing of their peaks. The STD-VH group reached its zenith at ZT18, while both CAF-VH and CAF-GSPE peaked at ZT22 and ZT21, respectively (Table 7). Regarding Catalase gene expression, only the CAF-VH group showed diurnal rhythmicity (*p* = 0.005), while both the STD-VH and CAF-GSPE groups showed a constant gene expression throughout the 24h (Figure 6C). In the case of *GPx1* gene expression (Figure 6D), the STD-VH group showed a diurnal rhythm (*p* = 0.005), while the CAF-GSPE treatment tended to restore the diurnal rhythmicity lost by the CAF (*p* = 0.097). Nevertheless, there were differences in the acrophases (Table 7). While the STD-VH group peaked at ZT6 (approximately 18 h after treatment), both CAF groups reached their zenith earlier, at ZT3 in the CAF-VH group and at ZT0 in the CAF-GSPE group. Additionally, similar to what was observed in ZT0-treated rats, diurnal rhythmicity in the expression of the *GSR* gene was not observed in either group (Figure 6E).

Remarkably, the expression of AOX genes did not show a clear differentiation between the daytime and nighttime phases of the day, as shown by the AOX enzymes and oxidative markers in the liver.

### 3.4. GSPE Treatment at Both ZT0 and ZT12 Partially Ameliorate Hepatic AOX Metabolic Profiles Altered Due to the CAF

Finally, to better understand the AOX metabolomic profile in both ZT0 and ZT12-treated rats, 25 AOX metabolites related to the direct and indirect AOX response, as well as the precursors of some AOX molecules such as GSH, were studied. The results are detailed in Tables S2–S4, and the liver AOX metabolomic profiles were further analyzed by heatmap analysis (Figures S1 and S2).

Regarding ZT0-treated rats, a total of nine metabolites showed significant differences, and four more showed tendencies, which included α-tocopherol, citric acid, glucose-6-phosphate, pyruvic acid, or taurine, among others (Table S3). In particular, the CAF increased the concentration of metabolites such as 4-hydroxyphenyllactic acid and citric acid at ZT1, α-tocopherol and isoleucine at ZT7, and 2-hydroxybutyric acid, 2-hydroxyisobutyric acid, α-tocopherol, and lactic acid at ZT13, suggesting that the state of oxidative stress in the CAF group would be more affected than in the CAF-GSPE group, likely due to obesity-induced oxidative stress, leading to alterations in the metabolomics profile (Figure S1). Interestingly, the CAF-GSPE group showed a decrease in the concentration of certain metabolites, such as serine at ZT1, phenylalanine at ZT7, and lactic acid, pyruvic acid, and 2-hydroxybutyric acid at ZT13, when comparing the CAF-VH and CAF-GSPE groups. This indicates an improvement in their metabolomic profile, bringing it closer to that presented by the STD-VH groups (Table S3).

**Figure 7:**
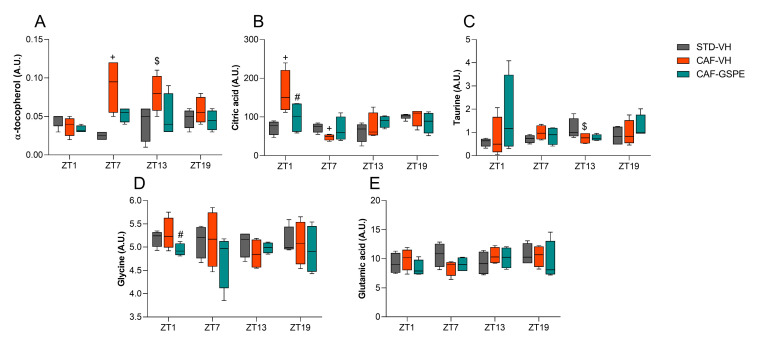
Hepatic levels of (**A**) α-tocopherol, (**B**) citric acid, (**C**) taurine, (**D**) glycine, and (**E**) glutamic acid of rats treated at ZT0. Data are shown as the median ± Min to max (*n* = 4). + indicates significant differences (*p* < 0.05) by diet effect (STD-VH vs. CAF-VH), $ indicates tendency (0.1 > *p* ≥ 0.05) by diet effect; # indicates tendency (0.1 > *p* ≥ 0.05) by treatment effect (CAF-VH vs. CAF-GSPE) using Mann–Whitney *U* test. STD, rats fed a Standard diet; CAF, rats fed a Cafeteria diet; VH, rats administered vehicle; GSPE, rats administered 25 mg/kg b.w. grape seed proanthocyanidin extract, A.U., arbitrary units.

Figure 7 shows the metabolomic levels of some of the most important AOX metabolites related to the AOX response. Specifically, the CAF increased α-tocopherol levels at ZT7 and ZT13 when compared to the STD-VH group. Meanwhile, GSPE treatment appeared to counteract the effects of the CAF, although the observed differences were not statistically significant. Regarding citric acid, it appears that the CAF induced an intermittent fluctuation of this metabolite during the light phase, increasing citric acid levels at ZT1 compared to the STD-VH group, while significantly decreasing the levels of this metabolite at ZT7 when compared to the same group. Taurine was also affected by the CAF, but only at ZT13, tending to decrease its levels when comparing the CAF-VH group to the STD-VH group. In the case of glycine and glutamic acid, the CAF had no influence on increasing or decreasing the levels of these metabolites. However, GSPE treatment tended to lower glycine levels at ZT1 when compared to the CAF-VH group.

Regarding ZT12-treated rats, a total of 11 metabolites showed significant differences, and two more showed tendencies (Table S4). Surprisingly, the heatmap analysis revealed a repetitive pattern in almost all the studied metabolites, with increased levels observed during the light phase (between ZT1 and ZT7) and decreased levels of them during the dark phase (between ZT13 and ZT19). This is the second time we have detected a repetitive day/night clustering, consistent with certain previously measured parameters, such as SOD or catalase (Figure S2). The metabolites that followed this pattern included fumaric acid, citric acid, and α-ketoglutaric acid, which are involved in the Krebs cycle, as well as amino acids such as leucine, glutamic acid, ornithine, isoleucine, and threonine. Moreover, GSPE treatment at ZT12 had an impact on several metabolites, increasing the levels of citric acid, α-tocopherol, 2-hydroxybutyric acid, glutamine, or fumaric acid at ZT19. Interestingly, GSPE treatment was able to modulate the concentration of metabolites, thereby approaching the levels obtained in the STD-VH. For instance, ornithine at ZT7 and glucose-6-phosphate, glutamine, glycine, glycolic acid, and serine at ZT13 showed improvement in the metabolomic profile of GSPE-treated rats (Table S4).

**Figure 8:**
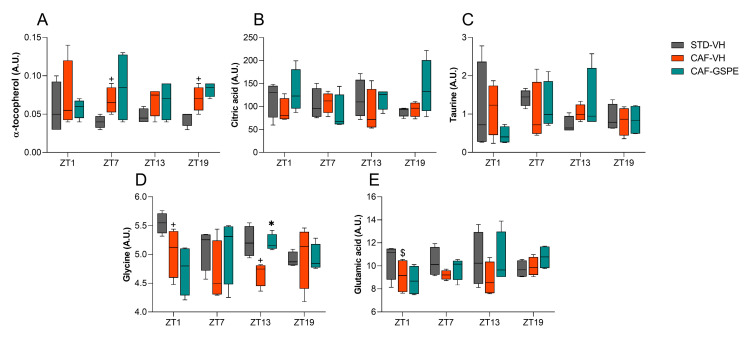
Hepatic levels of (**A**) α-tocopherol, (**B**) citric acid, (**C**) taurine, (**D**) glycine, and (**E**) glutamic acid of rats treated at ZT12. Data are shown as the median ± Min to max (*n* = 4). + indicates significant differences (*p* < 0.05) by diet effect (STD-VH vs. CAF-VH), $ indicates tendency (0.1 > *p* ≥ 0.05) by diet effect; * indicates significant differences (*p* < 0.05) by treatment effect (CAF-VH vs. CAF-GSPE) using Mann–Whitney *U* test. STD, rats fed a Standard diet; CAF, rats fed a Cafeteria diet; VH, rats administered vehicle; GSPE, rats administered 25 mg/kg b.w. grape seed proanthocyanidin extract, A.U., arbitrary units.

Figure 8 also showed that CAF significantly increased α-tocopherol levels at ZT7 and ZT19 when comparing the CAF-VH group with the STD-VH group. In contrast, citric acid and taurine did not show significant differences due to diet or treatment. However, the CAF did have an impact on glycine. In this case, the CAF-VH group showed significantly decreased levels of glycine when compared to the STD-VH group. Finally, GSPE treatment at ZT12 increased glycine levels at ZT13 compared to the CAF-VH group. Finally, glutamic acid levels were also affected by the CAF, decreasing their levels at ZT1 when comparing the CAF-VH group with the STD-VH group.

## 4. Discussion

Oxidative stress has been identified as an underlying factor mediating obesity and health issues associated with MetS. In an obesogenic context, systematic oxidative stress can be triggered through different biochemical mechanisms, such as the formation of the superoxide anion via the activation of NADPH oxidases, oxidative phosphorylation, glyceraldehyde autoxidation, and activation of protein kinase C, as well as polyol and hexamine signaling pathways [49,50]. Adipose tissue, in particular, is a major source of ROS production, as it disrupts mitochondrial function and leads to the formation of superoxide ions [50]. In addition, other factors related to an obesogenic diet, such as hyperleptinemia, reduced AOX defenses, chronic inflammation, or postprandial production of ROS, may contribute to oxidative stress. As a result, obesity can both result from and cause oxidative stress. [51]. In this context, AOX therapy using natural compounds in the diet, especially phenolic compounds, has been proven to be an effective and safe approach to addressing the consequences of ROS in obesity [52,53,54,55]. More concisely, the AOX activities of these compounds depend not only on their scavenger activity but also on their ability to prevent the activation of transcriptional factors such as NF-κB (nuclear factor κ-light-chain-enhancer of activated B cells) and reduce the expression of target genes, including those involved in inflammation [56,57,58,59,60].

In recent years, research has revealed that most physiological processes, including oxidation-reduction molecular states, follow a circadian rhythm that depends on the presence or absence of light, feeding–fasting, oxygen, and temperature cycles [61,62,63]. As mentioned above, obesity can lead to chronic metabolic disturbances that result in an imbalance in physiological oxidative processes. Thus, the consumption of natural products with AOX properties, including phenolic compounds, may play a role in restoring, at least partially, these altered physiological conditions. This, in turn, could lead to an improvement in obesity-associated pathologies, such as insulin resistance, diabetes, and hypercholesterolemia, among others [55,64,65,66,67].

Moreover, some recent findings have identified the molecular circadian machinery as an additional regulable target of phenolic compounds [68,69,70]. Taking this into account, the results of the present study explore the beneficial effects of a natural proanthocyanidin extract (GSPE) in an experimental model of circadian rhythm in rats euthanized at different time points to assess circadian rhythmicity in the liver oxidant system. Thus, we observed a clear circadian misalignment in certain ROS-related liver parameters due to the CAF. However, this misalignment could be restored by the oral administration of GSPE in a time-dependent manner, suggesting a potential chronotherapeutic effect of GSPE.

In this sense, as shown in Figure 2, GSPE administered at night (ZT12) reduces the body weight gain of the CAF-fed animals. This fact is accompanied by peaks in glucose, cholesterol, and triglycerides in the CAF-VH group during a specific period between ZT7 and ZT13 (Table 2), in which the GSPE treatment can notably reduce these parameters vs. CAF-VH (although not significantly). This effect is observed in both ZT0 and ZT12, but it is much more accentuated at ZT12, perhaps explained by the greater bioavailability of the GSPE or some of its components when administered at night. In this matter, our group has previously demonstrated that GSPE bioavailability was different when administered during the day than during the night [71]. Furthermore, it is worth mentioning that we also characterized the influence of gut microbiota on the seasonal differential effects of proanthocyanidins by at least modulating their bioavailability in diet-induced obese rats [35]. According to these findings, the gut microbiota has also been implicated in significantly impacting the time-of-day variation in the pharmacokinetics of drugs such as metformin, thus modulating host circadian rhythms and metabolic health [72,73]. Consequently, a plausible explanation for the chronotherapeutic behavior of GSPE could involve the day–night differential dynamics of the gut microbiota, which may differentially affect the GSPE bioavailability.

It is well known that the CAF (high sugar and fat content) causes an imbalance in ROS production and misalignment of the liver molecular machinery, leading to the disruption of the circadian system [74]. In our study, control rats (the STD-VH group) showed a higher AOX enzymatic profile in the activity phase (dark phase) compared to the resting phase (light phase) in both ZT0 and ZT12. Standard-fed rats also showed circadian rhythmicity in the activity of AOX enzymes and ROS-related biomarkers such as SOD, GPx1, GSH, and oxidized proteins (thiols), but not in Catalase at ZT0; and rhythm was present for SOD, GSH, and thiols, but not in Catalase and GPx1 at ZT12. These findings are consistent with previous studies that demonstrated a higher oxidative stress profile during the active phase [75,76]. Remarkably, in our study, the CAF delayed or induced the loss of this rhythmicity in GPx1, GSH, and thiols, which was partially or fully restored after GSPE treatment [67,68].

In addition, a recent study reported the use of AOX food supplements, including polyphenols, to retrain the circadian clock and adipose tissue function in humans. This therapeutic approach specifically targets the adipose clock, which plays a crucial role in maintaining the clock rhythm in an obesogenic context by regulating oxidative stress [77]. These findings align with the results of our study, where an increase in the activity of these AOX mediators was higher during the dark phase compared to the light phase, possibly reflecting the depletion of AOX molecules that counteract oxidative stress during the active phase of these animals. Furthermore, GSPE has been shown to act as a liver adaptation factor, enhancing the energetic profile and increasing mitochondrial function and oxidation during the dark phase, which is in accordance with our results on the activity of AOX mediators, including SOD, GPx1, and GSH during this period [78]. In particular, this chronotherapeutic feature of GSPE has also been shown through the acetylation of BMAL1, a key regulator of clock machinery, which exhibited a clear rhythm mainly during the active phase (dark period) [79]. Consistent with this, several models of obesity have shown that phenolic supplementation during the onset of the active phase may restore the functional dynamics of peripheral tissues during this period [30,70,80].

Findings from previous studies have shown that the CAF disrupts the TTFL genes of the central clock. For instance, Kohsaka et al. conducted a study in mice in which they described the effects of a high-fat diet on the diurnal rhythm of gene expression of the *Clock*, *Bmal1*, and *Per2* genes in both the hypothalamus and the liver after 6 weeks of a high-fat diet [8]. Their results showed that the *Clock* gene lost its diurnal rhythm in the hypothalamus and there was an attenuation in the amplitude of the *Bmal1* and *Per2* genes. In the liver, the high-fat diet caused a reduction in the amplitude of *Bmal1* expression in both light and dark periods, while the amplitude of *Per2* decreased only in the dark period. Similarly, Hsieh et al. also described the effects of a high-fat diet on mice livers [81]. They fed mice a high-fat diet for 11 months, resulting in the altered gene expression of both circadian clock genes and certain CCGs. In this sense, our results are in accordance with the circadian hepatic disruption induced by the CAF, regardless of the differences in administration time.

Furthermore, it has previously been shown that there are significant circadian variations in the transcript levels of AOX genes in the mouse liver and that they could affect physiological responses to oxidative stress at different times of the day [82,83,84]. Consistent with Xu et al., we were also able to assess the diurnal oscillations of some AOX-related genes in the liver, and, in parallel, our study revealed the influence of the CAF and the treatment with GSPE on the rhythmicity of those genes [82]. For example, in ZT0-treated rats, the *Sod2* expression in the STD-VH group showed rhythmicity, which was disrupted in the CAF-VH group. Importantly, GSPE treatment was able to recover the diurnal rhythm lost due to the CAF. In fact, GSPE treatment increased acrophase compared to the STD-VH group, suggesting that GSPE could improve the AOX response, especially at the mitochondrial level. Moreover, GSPE treatment also increased the acrophase of *Sod1* and brought the peak closer to that of the STD-VH group. Interestingly, a different effect was observed in ZT12-treated rats where *Sod1* and *Sod2* showed diurnal rhythmicity in all groups. However, GSPE treatment tended to restore the diurnal rhythmicity of *GPx1* lost by the CAF.

In this regard, other authors have also shown that the beneficial effect of bioactive compounds on the liver could be attributed in part to their ability to modulate circadian rhythms [31,78]. In this sense, Qi et al. demonstrated that tea polyphenols exert their beneficial effects on the oxidative status of hepatocytes by improving *Bmal1* expression [85]. More concisely, the authors showed that tea polyphenols behaved as a *Bmal1*-enhancing natural compound resulting in the alleviation of the redox imbalance by strengthening the KEAP1/NRF2 AOX defense pathway, and an amelioration of the mitochondrial dysfunction in a *Bmal1*-dependent manner. In agreement with these results, our group has previously described the time-of-day-dependent effect of GSPE on the diurnal rhythms of clock genes, as well as on mitochondrial dynamics and mitochondrial complex activity in the livers of both STD- and CAF-fed rats [70]. In more detail, treatment with GSPE at ZT0 restored the activity of the mitochondrial complexes, highly affected by the CAF, while GSPE at ZT12 was able to restore the diurnal rhythms of some clock genes such as *Bmal1* or *Rorα*, also altered by the CAF. Therefore, it is possible to speculate that the restoration of the diurnal rhythms of the clock genes by GSPE in the liver, as well as mitochondrial functionality, could also be related to the restoration of the diurnal rhythms of AOX enzyme activities, oxidative markers, and gene expression shown in our study, as circadian rhythms and oxidative stress are linked bidirectionally [86].

**Figure 9:**
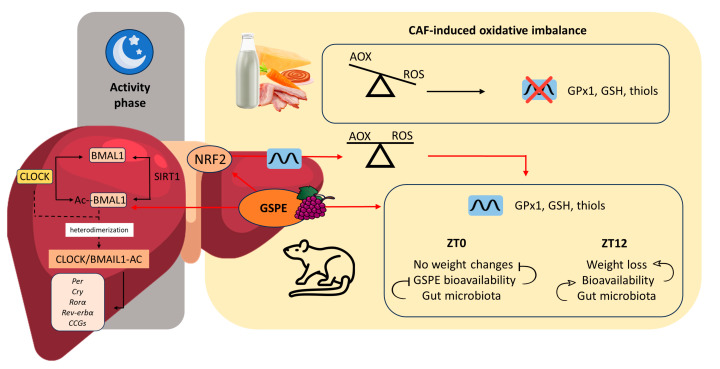
Potential mechanisms of action in which the chronotherapeutic effects of GSPE could be involved in the liver of diet-induced obese rats. The CAF induces an oxidative imbalance, thus enhancing ROS production, which leads to the loss of diurnal rhythmicity in certain AOX-related parameters such as GPx1, GSH, or thiols. However, oral GSPE intake has an influence on BMAL1 throughout its acetylation during the activity phase (dark phase in rats), contributing to restoring circadian rhythmicity in clock-controlled genes such as *Sod1* or *Nrf2*. The improvement in *Nrf2* expression mediated by BMAL1 contributes to alleviating the redox imbalance, reducing ROS, and achieving restoration of the diurnal rhythmicity of GPx1, GSH, and thiols. However, GSPE could also act as a chronotherapy, decreasing the body weight gain when administered at ZT12 (beginning of the active phase) but not when administered at ZT0 (resting phase), which could be mediated by the differential bioavailability of the extract depending on the time of administration and the differential day–night dynamics of the gut microbiota, which also influences GSPE bioavailability. BMAL1: Brain and muscle ARNT-Like 1; CLOCK: Circadian locomotor output cycles kaput; SIRT1: Sirtuin-1; *Per*: Period; *Cry*: Cryptochrome; *Rorα*: RAR-related orphan receptor alpha; *Rev-erbα*: Rev-Erb alpha; *CCGs*: Clock-controlled genes; NRF2: Nuclear factor erythroid 2-related factor 2; GSPE: Grape seed proanthocyanidin extract; AOX: Antioxidant; ROS: Reactive oxygen species; GPx1: Glutathione peroxidase 1; GSH: Glutathione; ZT: *Zeitgeber*. Arrowed lines: activation or induction; lines without an arrow: inhibition or negative regulation.

At this point, it is important to highlight that NRF2 regulates core and stabilizing circadian clock loops, which couples redox and timekeeping in mice [87]. In this sense, NRF2 and the clock machinery have been shown to comprise an interlocking loop that integrates cellular redox signals into tissue-specific circadian timekeeping. Consequently, NRF2 could be an important sensor and transducer of circadian rhythmicity, which, in view of our results, could also be postulated as an important factor to be taken into account for future experiments. Figure 9 shows some of the potential mechanisms that may be involved in certain effects caused by GSPE, including its chronotherapeutic effects, in an obesogenic context.

In order to deep dive into the chronotherapeutic profile of the GSPE treatment, we have also assessed the hepatic levels of some AOX-related metabolites and grouped them depending on the time of the extract administration (ZT0 or ZT12). In this aspect, the increase in some metabolites such as citric acid, isoleucine, or α-tocopherol in CAF-fed rats treated at both ZT0 and ZT12 suggest that the liver responds to the well-described pro-oxidative environment linked to obesity by attempting to enhance its AOX capacity [14,50,51]. Remarkably, GSPE administration at ZT0 reduced serine, phenylalanine, or pyruvic acid, among others. Conversely, its administration at ZT12 reduced ornithine, glutamine, or glucose-6-phosphate, thus improving the AOX profile and bringing it closer to the one observed in STD-VH animals. Additionally, GSPE treatment at ZT12 increased the metabolite concentration of glycine (at ZT13) and α-tocopherol, 2-hydroxybutyric acid, or glutamine (at ZT19), thereby enhancing the AOX response in a time-dependent manner. 

Consistent with our results, it has been widely demonstrated that patients with NAFLD exhibit reduced glycine circulating levels [88,89]. This pathological condition has been experimentally mitigated after glycine supplementation, which restores glycine levels and reduces oxidative stress through de novo GSH synthesis [90,91]. Similarly, the breakdown of glutamine, another GSH precursor, has been associated with fibrosis severity in mice models of steatohepatitis, and, in this context, the suppression of glutaminolysis inhibited the fibrotic response and disease progression [92]. Additionally, treatment with 2-hydroxybutyric acid has demonstrated protective effects in mice with acetaminophen-induced liver injury by decreasing the drug bioavailability and simultaneously increasing GSH levels [93]. Then, in the case of α-tocopherol (also known as Vitamin E), there is an increase in its hepatic concentration related to the CAF both at ZT0 and ZT12, which could be related to a hypothetical sequestration of vitamin E by the fat liver [94]. Moreover, in this case, GSPE treatment seemed to be more effective in “draining” this vitamin E from the liver at ZT0, which could be related to a more effective lipolytic effect by the proanthocyanidin extract at this time of administration.

Finally, we observed a day/night clustering pattern present in enzymatic activity, oxidative markers, and metabolomic results. Our analysis indicated that some of these parameters (GSH, thiol, etc.) were differentially modulated depending on the animal grouping (e.g., if the animals were grouped in the base of the light or the dark period). In this matter, it has been previously shown that night-shift workers exhibited lower levels of AOX defenses compared to day-shift workers [95]. Furthermore, in vertebrates, melatonin production is controlled by the circadian clock. Arylalkylamine N-acetyltransferase (AANAT) is the rate-limiting enzyme in melatonin biosynthesis, its expression is regulated at multiple levels, and these regulations are species-specific; different mechanisms operate in nocturnal and diurnal organisms. The mRNA level is regulated on both the transcriptional and post-transcriptional levels. Protein production is also regulated through internal ribosome entry site-mediated control of translation and posttranslational regulation of degradation [96]. Thus, it could be possible that the circadian clock-generated rhythms in melatonin may also contribute to the daily rhythms of AOX defense. Altogether, the above-mentioned evidence strengthens the circadian control of AOX systems and highlights the “biological rhythm of ROS” as an important factor to be considered for future chronotherapeutic dietetic AOX approaches.

Regarding the present study, although similar to several circadian studies [97,98,99], a significant limitation could be related to the low number of animals used (*n* = 4) for each death time-point (ZT1, ZT7, ZT13, and ZT19). This could decrease or hide some GSPE effects due to the statistical power. Nevertheless, this scientific design allows, at the same time, the assessment of the circadian rhythm of the liver AOX parameters in the cafeteria diet (both in GSPE-treated and non-treated animals), which is novel scientific information in the AOX research field. Regarding the GSPE effects, although this extract has been chemically characterized, it is a mixture of several procyanidins. In this sense, another limitation of the study might be that we have not yet identified the pure compound/s that is/are responsible for the observed beneficial effects of GSPE. This point opens future research perspectives, where several pure compounds (i.e., gallic acid, epicatechin, or procyanidin dimer) could be tested using the same experimental design and their effects on the circadian regulation of the AOX system can be compared between them. Finally, interesting future research could also be related to the effects of chronodisruption (social jetlag, sleep deprivation, etc.) and obesity, which are two main features of our present society, on the oxidative stress-related biological rhythms; and to also study if some part of the beneficial effects of AOXs (i.e., GSPE) could be hypothetically mediated by the reset or re-synchronization of the inner clock-machinery both at central and peripheral levels. 

## 5. Conclusions

Our study supports the idea that the AOX and circadian systems are interconnected by an equilibrium between peripheral tissues, including the liver, and the central clock. The misalignment of this equilibrium could induce disengagement between the peripheral and the central clock metabolism. In an obesity context, this fact can lead to the appearance of pathological conditions including a chronic redox imbalance. Previous studies by our research group have shown that the oral administration of GSPE was able to modulate clock gene expression in the liver, hypothalamus, and adipose tissue. Extending our previous results, in the present study, we have shown for the first time, to our knowledge, that GSPE can reduce oxidative stress induced by the CAF by retraining the circadian rhythm of AOX enzyme activities disrupted by the diet. Furthermore, we also observed a day-to-night effect of GSPE administration. Altogether, GSPE can be considered a chronotherapeutic agent capable to ameliorate the pathological underlying oxidative stress found in obesity and metabolic syndrome. These findings provide an explanation for its therapeutic benefits in other tissues, which also express rhythmicity and are conditioned by metabolic oxidative status.

## Figures and Tables

**Table 1:** Nucleotide sequences of primers used for RT-qPCR.

Gene	Accession Number	Forward Primer (5′ to 3′)	Reverse Primer (5′ to 3′)
*Sod1*	NM_017050.1	GGTGGTCCACGAGAAACAAG	CAATCACACCACAAGCCAAG
*Sod2*	NM_017051.2	AAGGAGCAAGGTCGCTTACA	ACACATCAATCCCCAGCAGT
*Catalase*	NM_012520.2	GAATGGCTATGGCTCACACA	CAAGTTTTTGATGCCCTGGT
*GPx1*	NM_030826.4	TGCAATCAGTTCGGACATC	CACCTCGCACTTCTCAAACA
*GSR*	NM_053906.2	ATCAAGGAGAAGCGGGATG	GCGTAGCCGTGGATGACT
*PPIA*	NM_017101.1	TCAAACACAAATGGTTCCCAGT	ATTCCTGGACCCAAAACGCT

**Table 2:** Serum biochemical parameters of STD- and CAF-fed rats treated at ZT0 or ZT12 with VH or 25 mg/kg b.w. GSPE.

			Glucose (mg/dL)	Triglycerides (mg/dL)	Cholesterol (mg/dL)	NEFA (mg/dL)
ZT0	ZT1	STD-VH CAF-VH CAF-GSPE	88.27 (87.04–91.34) 96.47 (91.64–101.04) 95.6 (94.07–106.31)	60.97 (56.62–68.42) 108.22 (100.66–116.06) + 105.26 (90.13–123.62)	97.26 (81.37–106.85) 93.83 (88.1–101.71) 95.82 (92.26–106.46)	19.72 (17.23–24.42) 22.87 (21.66–25.92) 21.81 (20.27–22.31)
	ZT7	STD-VH CAF-VH CAF-GSPE	86.99 (86.13–90.32) 112.14 (105.06–117.92) + 116.47 (111.27–119.8)	102.7 (102.16–104.32) 331.89 (312.97–338.11) $ 276.22 (251.62–304.86)	103.11 (96.69–107.59) 128.02 (120.62–141.63) 141.25 (119.07–164.2)	31.44 (29.57–34.28) 29.81 (28.8–32.16) 34.13 (32.31–35)
	ZT13	STD-VH CAF-VH CAF-GSPE	86.67 (84.78–91.5) 113.58 (106.59–118.28) $ 99.12 (94.21–101.03)	54.96 (40–68.09) 184.23 (154.72–234.45) + 103.95 (98.17–110.53)	91.5 (83.67–95.44) 97.58 (81.47–128.97) 76.53 (69.16–86.97)	25.64 (15.8–35.22) 31.13 (28.05–35.71) 36.27 (35–36.78)
	ZT19	STD-VH CAF-VH CAF-GSPE	77.79 (74.75–82.66) 100.9 (94.29–102.81) 102.09 (83.87–115.6)	51.95 (43.94–58.22) 143.42 (125.17–151.93) + 131.83 (129.07–133.02)	85.27 (69.73–101.29) 94.01 (91.06–100) 81.19 (71.79–91.94)	22.47 (19.07–25.64) 27.19 (26.65–29.21) 29.85 (28.54–33.23)
ZT12	ZT1	STD-VH CAF-VH CAF-GSPE	90.75 (90.24–91.04) 101.81 (97.34–108.29) $ 117.61 (116.87–117.75) #	74.69 (56.62–102.3) 115.13 (110.64–127.41) 181.41 (141.95–221.28)	128.63 (104.17–144.09) 92.74 (84.58–96.55) 95.6 (87.07–101.21)	23.7 (20.27–34.9) 26.41 (22.82–30.18) 25.82 (20.65–35.82)
	ZT7	STD-VH CAF-VH CAF-GSPE	90.17 (78.03–99.42) 113.29 (110.84–118.79) + 123.12 (116.91–132.08)	160.54 (137.3–186.76) 302.7 (278.38–344.59) + 338.38 (302.16–402.97)	123.74 (114.79–143.19) 111.28 (104.09–129.57) 145.14 (130.93–165.56)	29.52 (26.3–33.37) 31.25 (30.19–33.8) 34.71 (28.03–41.15)
	ZT13	STD-VH CAF-VH CAF-GSPE	83.88 (78.75–90.62) 124.38 (108.65–139.85) + 108.81 (100.02–121.72)	51.01 (47.89–52.96) 219.64 (177.75–230.92) + 145.14 (127.84–200.93)	102.52 (91.99–111.03) 118.99 (110.34–127.92) 106.86 (102.63–122.46)	27.56 (24.26–30.59) 33.89 (29.45–37.72) 32.25 (30.4–36.08)
	ZT19	STD-VH CAF-VH CAF-GSPE	79.61 (74.19–86.42) 119.94 (112.05–132.94) + 95.22 (89.4–112.17)	65.48 (60.47–70.25) 211.27 (149.74–275.05) + 146.16 (126.32–199.72)	88.8 (86.05–96.3) 133.02 (104.78–162.27) 112 (94.62–145.82)	30.96 (28.13–33.85) 30.76 (27.34–33.6) 24.11 (22.67–25.69)

Values are expressed as the median (Q1-Q3), (*n* = 3–4) for ZT0 and ZT12 conditions. + indicates significant differences (*p* < 0.05) by diet effect (STD-VH vs. CAF-VH, $ indicates tendency (0.1 > *p* ≥ 0.05) by diet effect; # indicates tendency (0.1 > *p* ≥ 0.05) by treatment effect (CAF-VH vs. CAF-GSPE) using Mann–Whitney *U* test. STD, rats fed a Standard diet; CAF, rats fed a Cafeteria diet; VH, rats administered vehicle; GSPE, rats administered 25 mg/kg b.w. grape seed proanthocyanidin extract.

**Table 3:** Circadian parameters and diurnal oscillations estimated for liver AOX-related parameters in ZT0-treated rats.

Parameter	Group	Period (h)	*p*	MESOR	Amplitude	*p* (Amplitude)	Acrophase (h)	*p* (Acrophase)
SOD	STD-VH CAF-VH CAF-GSPE	24 24 24	0.001 ** 0.002 ** <0.001 ***	11.52 12.30 12.29	2.52 1.85 3.62	<0.001 *** <0.001 *** <0.001 ***	15.99 15.59 15.08	<0.001 *** <0.001 *** <0.001 ***
Catalase	STD-VH CAF-VH CAF-GSPE	24 24 24	0.531 0.697 0.088 #	709.56 714.29 676.71	24.36 15.90 55.97	0.249 0.389 0.014 *	16.83 6.27 11.63	<0.001 *** <0.001 *** <0.001 ***
GPx1	STD-VH CAF-VH CAF-GSPE	24 24 24	0.027 * 0.033 * 0.024 *	2271.60 1897.17 1847.25	278.79 290.64 425.05	0.002 ** 0.003 ** 0.001 **	16.02 19.60 15.09	<0.001 *** <0.001 *** <0.001 ***
GSH	STD-VH CAF-VH CAF-GSPE	24 24 24	0.001 ** 0.434 0.063 #	2583.39 2021.05 1744.49	591.45 255.83 396.18	<0.001 *** 0.181 0.008 **	4.04 6.95 5.44	<0.001 *** 0.023 * <0.001 ***
Thiols	STD-VH CAF-VH CAF-GSPE	24 24 24	0.039 * 0.082 # 0.052 #	94.85 92.03 94.49	17.32 12.53 15.92	0.004 ** 0.013 * 0.006 **	2.78 6.67 6.84	0.034 * <0.001 *** <0.001 ***

MESOR is an average value adjusted for diurnal rhythm, the amplitude is the difference between a wave’s peak and average, and the acrophase is the time when the oscillation peak occurs. The values represent the estimated circadian parameters determined by the Cosinor method. * indicates the significant detection (*p* < 0.05) of diurnal oscillations and parameters, ** indicates *p* < 0.01, *** indicates *p* < 0.001; # indicates tendency (0.1 > *p* ≥ 0.05). STD, rats fed a Standard diet; CAF, rats fed a Cafeteria diet; VH, rats administered vehicle; GSPE, rats administered 25 mg/kg b.w. grape seed proanthocyanidin extract; h, hours.

**Table 4:** Circadian parameters and diurnal oscillations estimated for liver AOX-related parameters in ZT12-treated rats.

Parameter	Group	Period (h)	*p*	MESOR	Amplitude	*p* (Amplitude)	Acrophase (h)	*p* (Acrophase)
SOD	STD-VH CAF-VH CAF-GSPE	24 24 24	0.010 * 0.055 # 0.014 *	12.05 12.04 11.45	1.66 1.34 1.05	<0.001 *** <0.001 *** <0.001 ***	15.31 17.36 16.96	<0.001 *** <0.001 *** <0.001 ***
Catalase	STD-VH CAF-VH CAF-GSPE	24 24 24	0.345 0.045 * 0.061 #	697.89 687.95 693.49	26.90 56.24 46.33	0.128 0.004 ** 0.008 **	4.21 4.80 5.10	0.091 # 0.001 ** <0.001 ***
GPx1	STD-VH CAF-VH CAF-GSPE	24 24 24	0.393 0.729 0.993	2328.48 1770.59 1751.78	143.67 74.43 15.10	0.156 0.421 0.906	11.79 20.32 12.64	<0.001 *** <0.001 *** 0.696
GSH	STD-VH CAF-VH CAF-GSPE	24 24 24	0.020 * 0.189 0.172	2487.20 2074.93 1884.68	600.67 319.96 298.25	0.001 ** 0.051 # 0.045 *	4.74 4.80 3.83	<0.001 *** 0.014 * 0.044 *
Thiols	STD-VH CAF-VH CAF-GSPE	24 24 24	0.009 ** <0.001 *** 0.001 **	104.30 105.56 108.25	21.19 31.44 25.55	<0.001 *** <0.001 *** <0.001 ***	5.25 4.23 3.63	<0.001 *** <0.001 *** <0.001 ***

MESOR is an average value adjusted for diurnal rhythm, the amplitude is the difference between a wave’s peak and average, and the acrophase is the time when the oscillation peak occurs. The values represent the estimated circadian parameters determined by the Cosinor method. * indicates the significant detection (*p* < 0.05) of diurnal oscillations and parameters, ** indicates *p* < 0.01, *** indicates *p* < 0.001; # indicates tendency (0.1 > *p* ≥ 0.05). STD, rats fed a Standard diet; CAF, rats fed a Cafeteria diet; VH, rats administered vehicle; GSPE, rats administered 25 mg/kg b.w. grape seed proanthocyanidin extract; h, hours.

**Table 5:** Liver AOX-related parameters of STD- and CAF-fed rats treated at ZT0 or ZT12 with VH or 25 mg/kg b.w. GSPE clustering by day/night sample-obtention.

			SOD (U SOD/mg Prot)	Catalase (U/mg Prot)	GPx1 (mU GPx1/mg Prot)	GSH (nmol GSH/mg Prot)	Oxidized Proteins (nmol -SH/mg Prot)
ZT0	LIGHT PHASE	STD-VH CAF-VH CAF-GSPE	9.67 (9.3–10.26) 10.88 (10.42–11.66) ++ 9.87 (9.58–10.3) **	668.13 (664.70–758.88) 720.95 (691.91–776.14) 642.71 (634.64–678.85) *	2078.03 (2007.69–2115.74) 1741.21 (1424.32–1995.01) 1410.08 (1302.11–1831.33)	3057.76 (2989.45–3207.91) 1998.10 (1828.45–2923.07) + 2054.13 (1726.91–2317.81)	115.98 (90.38–118.84) 93.71 (87.57–120.04) 105.40 (87.03–118.47)
	DARK PHASE	STD-VH CAF-VH CAF-GSPE	13.11 (12.73–14.35) && 13.75 (12.62–15.03) && 14.39 (13.67–15.81) &&	722.30 (709.01–748.09) 700.33 (687.36–724.93) 676.06 (648.30–706.40)	2494.95 (2365.07–2599.67) & 2013.04 (1891.08–2191.33) ++ 2216.50 (1786.01–2372.41) &	2167.02 (2065.72–2255.08) && 1862.04 (1633.34–2119.29) 1495.00 (1360.02–1699.41) &	83.76 (78.48–87.65) & 83.69 (79.84–87.68) & 82.92 (78.59–88.16) &
ZT12	LIGHT PHASE	STD-VH CAF-VH CAF-GSPE	10.64 (10.02–11.62) 11.13 (10.58–11.67) 10.85 (10.33–11.04)	725.53 (698.82–744.47) 717.64 (655.40–740.22) 740.96 (686.92–766.97)	2296.09 (2125.45–2481.63) 1742.62 (1617.97–1920.80) ++ 1806.08 (1607.78–1876.91)	3005.38 (2741.25–3079.27) 2414.31 (2110.62–2563.82) + 2137.81 (1923.12–2213.25)	112.93 (109.39–124.75) 130.16 (118.01–135.57) 129.61 (114.16–137.15)
	DARK PHASE	STD-VH CAF-VH CAF-GSPE	13.39 (12.17–13.82) && 12.66 (12.19–13.46) & 12.57 (11.24–12.9) &	662.92 (651.74–690.47) & 647.61 (625.13–668.53) & 656.97 (644.57–679.19)	2449.82 (2319.24–2529.99) 1767.47 (1636.02–1882.40) ++ 1790.96 (1496.10–2108.56)	1989.62 (1884.67–2420.08) & 1941.43 (1560.06–2069.69) & 1651.59 (1451.46- 1835.57)	89.78 (78.92–100.80) && 82.24 (79.21–89.49) &&& 89.53 (77.87–93.04) &&

Values are expressed as the median (Q1-Q3), (*n* = 7–8) for ZT0 and ZT12 conditions. + indicates significant differences (*p* < 0.05) by diet effect (STD-VH vs. CAF-VH), ++ indicates *p* < 0.01 by diet effect * indicates significant differences (*p* < 0.05) by treatment effect (CAF-VH vs. CAF-GSPE), ** indicates *p* < 0.01 by treatment effect, & indicates significant differences (*p* < 0.05) by daylight effect (light vs. dark phases), && indicates *p* < 0.01 by daylight effect, &&& indicates *p* < 0.001 by daylight effect using Mann–Whitney *U* test. STD, rats fed a Standard diet; CAF, rats fed a Cafeteria diet; VH, rats administered vehicle; GSPE, rats administered 25 mg/kg b.w. grape seed proanthocyanidin extract.

**Table 6:** Circadian parameters and diurnal oscillations estimated of liver AOX-related genes in ZT0-treated rats.

Parameter	Group	Period (h)	*p*	MESOR	Amplitude	*p* (Amplitude)	Acrophase (h)	*p* (Acrophase)
*Sod1*	STD-VH CAF-VH CAF-GSPE	24 24 24	0.643 0.266 0.021 *	0.98 1.02 1.04	0.08 0.11 0.13	0.339 0.086 # 0.001 **	22.97 15.35 20.33	<0.001 *** <0.001 *** <0.001 ***
*Sod2*	STD-VH CAF-VH CAF-GSPE	24 24 24	0.008 ** 0.146 0.001 **	1.05 1.27 1.34	0.31 1.27 1.34	<0.001 *** 0.034 * <0.001 ***	19.59 18.76 19.84	<0.001 *** <0.001 *** <0.001 ***
*Catalase*	STD-VH CAF-VH CAF-GSPE	24 24 24	0.891 0.170 0.911	1.04 1.04 0.98	0.04 0.12 0.04	0.629 0.044 * 0.666	14.09 13.43 22.68	0.075 # <0.001 *** 0.009 **
*GPx1*	STD-VH CAF-VH CAF-GSPE	24 24 24	0.367 0.966 0.557	0.92 0.86 0.87	0.12 0.02 0.07	0.141 0.793 0.268	1.77 19.93 22.55	0.494 0.172 <0.001 ***
*GSR*	STD-VH CAF-VH CAF-GSPE	24 24 24	0.176 0.943 0.746	1.07 1.14 1.11	0.14 0.03 0.07	0.046 * 0.731 0.439	18.37 11.12 19.40	<0.001 *** <0.001 *** <0.001 ***

MESOR is an average value adjusted for diurnal rhythm, the amplitude is the difference between a wave’s peak and average, and the acrophase is the time when the oscillation peak occurs. The values represent the estimated circadian parameters determined by the Cosinor method. * indicates the significant detection (*p* < 0.05) of diurnal oscillations and parameters, ** indicates *p* < 0.01, *** indicates *p* < 0.001; # indicates tendency (0.1 > *p* ≥ 0.05). STD, rats fed a Standard diet; CAF, rats fed a Cafeteria diet; VH, rats administered vehicle; GSPE, rats administered 25 mg/kg b.w. grape seed proanthocyanidin extract; h, hours.

**Table 7:** Circadian parameters and diurnal oscillations estimated for liver AOX-related genes in ZT12-treated rats.

Parameter	Group	Period (h)	*p*	MESOR	Amplitude	*p* (Amplitude)	Acrophase (h)	*p* (Acrophase)
*Sod1*	STD-VH CAF-VH CAF-GSPE	24 24 24	0.095 # 0.047 * 0.084 #	0.86 0.83 0.74	0.24 0.24 0.16	0.017 * 0.005 ** 0.014 *	19.45 19.70 19.49	<0.001 *** <0.001 *** <0.001 ***
*Sod2*	STD-VH CAF-VH CAF-GSPE	24 24 24	0.003 ** 0.008 ** 0.002 **	1.12 1.35 1.12	0.38 0.42 0.34	<0.001 *** <0.001 *** <0.001 ***	18.40 20.81 20.28	<0.001 *** <0.001 *** <0.001 ***
*Catalase*	STD-VH CAF-VH CAF-GSPE	24 24 24	0.432 0.005 ** 0.309	1.20 1.18 0.95	0.09 0.18 0.09	0.180 <0.001 *** 0.109	11.89 21.24 21.93	<0.001 *** <0.001 *** <0.001 ***
*GPx1*	STD-VH CAF-VH CAF-GSPE	24 24 24	0.005 ** 0.100 0.097 #	1.05 1.12 0.86	0.32 0.17 0.11	<0.001 *** 0.019 * 0.018 *	6.25 2.77 0.34	<0.001 *** 0.088# 0.831
*GSR*	STD-VH CAF-VH CAF-GSPE	24 24 24	0.239 0.216 0.477	1.14 1.15 0.97	0.14 0.15 0.08	0.074 # 0.063 # 0.210	17.05 21.17 20.10	<0.001 *** <0.001 *** <0.001 ***

MESOR is an average value adjusted for diurnal rhythm, the amplitude is the difference between a wave’s peak and average, and the acrophase is the time when the oscillation peak occurs. The values represent the estimated circadian parameters determined by the Cosinor method. * indicates the significant detection (*p* < 0.05) of diurnal oscillations and parameters, ** indicates *p* < 0.01, *** indicates *p* < 0.001; # indicates tendency (0.1 > *p* ≥ 0.05). STD, rats fed a Standard diet; CAF, rats fed a Cafeteria diet; VH, rats administered vehicle; GSPE, rats administered 25 mg/kg b.w. grape seed proanthocyanidin extract; h, hours.

## Data Availability

The data presented in this study are available upon request from the corresponding author. The data are not publicly available due to the lack of a platform on which to publish them.

## Supplementary Materials

The following supporting information can be downloaded at: https://www.mdpi.com/article/10.3390/antiox12081606/s1, Table S1: Weekly food intake of STD- and CAF-fed rats treated at ZT0 or ZT12 with VH or 25 mg/kg b.w. GSPE expressed in g/day and kcal/day; Table S2: Liver AOX-related parameters of STD- and CAF-fed rats treated at ZT0 or ZT12 with VH or 25 mg/kg b.w. GSPE; Table S3: AOX-related metabolites in ZT0-treated rats expressed in arbitrary units; Table S4: AOX-related metabolites in ZT12-treated rats expressed in arbitrary units; Figure S1: Heatmap analysis of AOX-related detected metabolites in liver metabolomics of rats treated at ZT0; Figure S2: Heatmap analysis of AOX-related detected metabolites in liver metabolomics of rats treated at ZT12.

## References

[1] Sanford A.B.A., da Cunha L.S., Machado C.B., de Pinho Pessoa F.M.C., Silva A.N.d.S., Ribeiro R.M., Moreira F.C., de Moraes Filho M.O., de Moraes M.E.A., de Souza L.E.B. (2022). Circadian Rhythm Dysregulation and Leukemia Development: The Role of Clock Genes as Promising Biomarkers. Int. J. Mol. Sci..

[2] Sato T., Sassone-Corsi P. (2022). Nutrition, Metabolism, and Epigenetics: Pathways of Circadian Reprogramming. EMBO Rep..

[3] Neves A.R., Albuquerque T., Quintela T., Costa D. (2022). Circadian Rhythm and Disease: Relationship, New Insights, and Future Perspectives. J. Cell Physiol..

[4] Albrecht U. (2017). The Circadian Clock, Metabolism and Obesity. Obes. Rev..

[5] Johnston J.D., Ordovás J.M., Scheer F.A., Turek F.W. (2016). Circadian Rhythms, Metabolism, and Chrononutrition in Rodents and Humans. Adv. Nutr..

[6] Li Y., Ma J., Yao K., Su W., Tan B., Wu X., Huang X., Li T., Yin Y., Tosini G. (2020). Circadian Rhythms and Obesity: Timekeeping Governs Lipid Metabolism. J. Pineal Res..

[7] Salgado-Delgado R.C., Saderi N., Basualdo M.d.C., Guerrero-Vargas N.N., Escobar C., Buijs R.M. (2013). Shift Work or Food Intake during the Rest Phase Promotes Metabolic Disruption and Desynchrony of Liver Genes in Male Rats. PLoS ONE.

[8] Kohsaka A., Laposky A.D., Ramsey K.M., Estrada C., Joshu C., Kobayashi Y., Turek F.W., Bass J. (2007). High-Fat Diet Disrupts Behavioral and Molecular Circadian Rhythms in Mice. Cell Metab..

[9] Laermans J., Depoortere I. (2016). Chronobesity: Role of the Circadian System in the Obesity Epidemic. Obes. Rev..

[10] Saklayen M.G. (2018). The Global Epidemic of the Metabolic Syndrome. Curr. Hypertens. Rep..

[11] McCracken E., Monaghan M., Sreenivasan S. (2018). Pathophysiology of the Metabolic Syndrome. Clin. Dermatol..

[12] Bovolini A., Garcia J., Andrade M.A., Duarte J.A. (2021). Metabolic Syndrome Pathophysiology and Predisposing Factors. Int. J. Sports Med..

[13] Tanianskii D.A., Jarzebska N., Birkenfeld A.L., O’sullivan J.F., Rodionov R.N. (2019). Beta-Aminoisobutyric Acid as a Novel Regulator of Carbohydrate and Lipid Metabolism. Nutrients.

[14] Rani V., Deep G., Singh R.K., Palle K., Yadav U.C.S. (2016). Oxidative Stress and Metabolic Disorders: Pathogenesis and Therapeutic Strategies. Life Sci..

[15] Ali S.S., Ahsan H., Zia M.K., Siddiqui T., Khan F.H. (2020). Understanding Oxidants and AOXs: Classical Team with New Players. J. Food Biochem..

[16] Kondratov R.V., Vykhovanets O., Kondratova A.A., Antoch M.P. (2009). AOX N-Acetyl-L-Cysteine Ameliorates Symptoms of Premature Aging Associated with the Deficiency of the Circadian Protein BMAL1. Aging.

[17] Chhunchha B., Kubo E., Singh D.P. (2020). Clock Protein Bmal1 and Nrf2 Cooperatively Control Aging or Oxidative Response and Redox Homeostasis by Regulating Rhythmic Expression of Prdx6. Cells.

[18] Da Porto A., Cavarape A., Colussi G., Casarsa V., Catena C., Sechi L.A. (2021). Polyphenols Rich Diets and Risk of Type 2 Diabetes. Nutrients.

[19] Deledda A., Annunziata G., Tenore G.C., Palmas V., Manzin A., Velluzzi F. (2021). Diet-Derived AOXs and Their Role in Inflammation, Obesity and Gut Microbiota Modulation. AOXs.

[20] Ferramosca A., Di Giacomo M., Zara V. (2017). AOX Dietary Approach in Treatment of Fatty Liver: New Insights and Updates. World J. Gastroenterol..

[21] Kwaśniewska M., Pikala M., Grygorczuk O., Waśkiewicz A., Stepaniak U., Pająk A., Kozakiewicz K., Nadrowski P., Zdrojewski T., Puch-Walczak A. (2023). Dietary AOXs, Quality of Nutrition and Cardiovascular Characteristics among Omnivores, Flexitarians and Vegetarians in Poland—The Results of Multicenter National Representative Survey WOBASZ. AOXs.

[22] Rahman M.M., Rahaman M.S., Islam M.R., Rahman F., Mithi F.M., Alqahtani T., Almikhlafi M.A., Alghamdi S.Q., Alruwaili A.S., Hossain M.S. (2022). Role of Phenolic Compounds in Human Disease: Current Knowledge and Future Prospects. Molecules.

[23] Cannataro R., Fazio A., Torre C.L., Caroleo M.C., Cione E. (2021). Polyphenols in the Mediterranean Diet: From Dietary Sources to MicroRNA Modulation. AOXs.

[24] Serrano J., Casanova-Martí À., Gil-Cardoso K., Blay M.T., Terra X., Pinent M., Ardévol A. (2016). Acutely Administered Grape-Seed Proanthocyanidin Extract Acts as a Satiating Agent. Food Funct..

[25] Unusan N. (2020). Proanthocyanidins in Grape Seeds: An Updated Review of Their Health Benefits and Potential Uses in the Food Industry. J. Funct. Foods.

[26] Montagut G., Bladé C., Blay M., Fernández-Larrea J., Pujadas G., Salvadó M.J., Arola L., Pinent M., Ardévol A. (2010). Effects of a Grapeseed Procyanidin Extract (GSPE) on Insulin Resistance. J. Nutr. Biochem..

[27] Pons Z., Margalef M., Bravo F.I., Arola-Arnal A., Muguerza B. (2017). Chronic Administration of Grape-Seed Polyphenols Attenuates the Development of Hypertension and Improves Other Cardiometabolic Risk Factors Associated with the Metabolic Syndrome in Cafeteria Diet-Fed Rats. Br. J. Nutr..

[28] Puiggròs F., Llópiz N., Ardévol A., Bladé C., Arola L., Salvadó M.J. (2005). Grape Seed Procyanidins Prevent Oxidative Injury by Modulating the Expression of AOX Enzyme Systems. J. Agric. Food Chem..

[29] Liu M., Yun P., Hu Y., Yang J., Khadka R.B., Peng X. (2020). Effects of Grape Seed Proanthocyanidin Extract on Obesity. Obes. Facts.

[30] Ribas-Latre A., Del Bas J.M., Baselga-Escudero L., Casanova E., Arola-Arnal A., Salvadó M.J., Arola L., Bladé C. (2015). Dietary Proanthocyanidins Modulate Melatonin Levels in Plasma and the Expression Pattern of Clock Genes in the Hypothalamus of Rats. Mol. Nutr. Food Res..

[31] Ribas-Latre A., Baselga-Escudero L., Casanova E., Arola-Arnal A., Salvadó M.J., Bladé C., Arola L. (2015). Dietary Proanthocyanidins Modulate BMAL1 Acetylation, Nampt Expression and NAD Levels in Rat Liver. Sci. Rep..

[32] Gadacha W., Ben-Attia M., Bonnefont-Rousselot D., Aouani E., Ghanem-Boughanmi N., Touitou Y. (2009). Resveratrol Opposite Effects on Rat Tissue Lipoperoxidation: Pro-Oxidant during Day-Time and AOX at Night. Redox Rep..

[33] Arreaza-Gil V., Escobar-Martínez I., Mulero M., Muguerza B., Suárez M., Arola-Arnal A., Torres-Fuentes C. (2023). Gut Microbiota Influences the Photoperiod Effects on Proanthocyanidins Bioavailability in Diet-Induced Obese Rats. Mol. Nutr. Food Res..

[34] Sampey B.P., Vanhoose A.M., Winfield H.M., Freemerman A.J., Muehlbauer M.J., Fueger P.T., Newgard C.B., Makowski L. (2011). Cafeteria Diet Is a Robust Model of Human Metabolic Syndrome with Liver and Adipose Inflammation: Comparison to High-Fat Diet. Obesity.

[35] Arreaza-Gil V., Escobar-Martínez I., Muguerza B., Aragonès G., Suárez M., Torres-Fuentes C., Arola-Arnal A. (2022). The Effects of Grape Seed Proanthocyanidins in Cafeteria Diet-Induced Obese Fischer 344 Rats Are Influenced by Faecal Microbiota in a Photoperiod Dependent Manner. Food Funct..

[36] Zeeni N., Dagher-Hamalian C., Dimassi H., Faour W.H. (2015). Cafeteria Diet-Fed Mice Is a Pertinent Model of Obesity-Induced Organ Damage: A Potential Role of Inflammation. Inflamm. Res..

[37] Aragonès G., Suárez M., Ardid-Ruiz A., Vinaixa M., Rodríguez M.A., Correig X., Arola L., Bladé C. (2016). Dietary Proanthocyanidins Boost Hepatic NAD+ Metabolism and SIRT1 Expression and Activity in a Dose-Dependent Manner in Healthy Rats. Sci. Rep..

[38] Reagan-Shaw S., Nihal M., Ahmad N. (2008). Dose Translation from Animal to Human Studies Revisited. FASEB J..

[39] Livak K.J., Schmittgen T.D. (2001). Analysis of Relative Gene Expression Data Using Real-Time Quantitative PCR and the 2-ΔΔCT Method. Methods.

[40] Marklund S. (1976). Spectrophotometric Study of Spontaneous Disproportionation of Superoxide Anion Radical and Sensitive Direct Assay for Superoxide Dismutase. J. Biol. Chem..

[41] Beers R., Sizer I. (1952). A Spectrophotometric Method for Measuring the Breakdown of Hydrogen Peroxide by Catalase. J. Biol. Chem..

[42] Flohé L., Günzler W.A. (1984). Assays of Glutathione Peroxidase. Methods Enzym..

[43] Griffith O.W. (1980). Determination of Glutathione and Glutathione Disulfide Using Glutathione Reductase and 2-Vinylpyridine. Anal. Biochem..

[44] Faure P., Lafond J.-L. (1995). Measurement of Plasma Sulfhydryl and Carbonyl Groups as a Possible Indicator of Protein Oxidation. Analysis of Free Radicals in Biological Systems.

[45] Witko-Sarsat V., Friedlander M., Nguyen Khoa T., Capeillère-Blandin C., Nguyen A.T., Canteloup S., Dayer J.M., Jungers P., Drüeke T., Descamps-Latscha B. (1998). Advanced Oxidation Protein Products as Novel Mediators of Inflammation and Monocyte Activation in Chronic Renal Failure. J. Immunol..

[46] Cajka T., Fiehn O. (2016). Toward Merging Untargeted and Targeted Methods in Mass Spectrometry-Based Metabolomics and Lipidomics. Anal. Chem..

[47] Xia J., Wishart D.S. (2011). Web-Based Inference of Biological Patterns, Functions and Pathways from Metabolomic Data Using MetaboAnalyst. Nat. Protoc..

[48] Moškon M. (2020). CosinorPy: A Python Package for Cosinor-Based Rhythmometry. BMC Bioinform..

[49] Savini I., Catani M., Evangelista D., Gasperi V., Avigliano L. (2013). Obesity-Associated Oxidative Stress: Strategies Finalized to Improve Redox State. Int. J. Mol. Sci..

[50] Marseglia L., Manti S., D’Angelo G., Nicotera A., Parisi E., Di Rosa G., Gitto E., Arrigo T. (2014). Oxidative Stress in Obesity: A Critical Component in Human Diseases. Int. J. Mol. Sci..

[51] Manna P., Jain S.K. (2015). Obesity, Oxidative Stress, Adipose Tissue Dysfunction, and the Associated Health Risks: Causes and Therapeutic Strategies. Metab. Syndr. Relat. Disord..

[52] Moliner C., Núñez S., Cásedas G., Valero M.S., Dias M.I., Barros L., López V., Gómez-Rincón C. (2023). Flowers of *Allium cepa* L. as Nutraceuticals: Phenolic Composition and Anti-Obesity and AOX Effects in Caenorhabditis Elegans. AOXs.

[53] Wu G., Cheng H., Guo H., Li Z., Li D., Xie Z. (2023). Tea Polyphenol EGCG Ameliorates Obesity-Related Complications by Regulating Lipidomic Pathway in Leptin Receptor Knockout Rats. J. Nutr. Biochem..

[54] Delgadillo-Puga C., Sánchez-Castillo D.R., Cariño-Cervantes Y.Y., Torre-Villalvazo I., Tovar-Palacio C., Vásquez-Reyes S., Furuzawa-Carballeda J., Acevedo-Carabantes J.A., Camacho-Corona M.d.R., Guzmán-Mar J.L. (2023). Vachellia Farnesiana Pods or a Polyphenolic Extract Derived from Them Exert Immunomodulatory, Metabolic, Renoprotective, and Prebiotic Effects in Mice Fed a High-Fat Diet. Int. J. Mol. Sci..

[55] Maturana G., Segovia J., Olea-Azar C., Uribe-Oporto E., Espinosa A., Zúñiga-López M.C. (2023). Evaluation of the Effects of Chia (*Salvia hispanica* L.) Leaves Ethanolic Extracts Supplementation on Biochemical and Hepatic Markers on Diet-Induced Obese Mice. AOXs.

[56] Pérez-Torres I., Castrejón-Téllez V., Soto M.E., Rubio-Ruiz M.E., Manzano-Pech L., Guarner-Lans V. (2021). Oxidative Stress, Plant Natural AOXs, and Obesity. Int. J. Mol. Sci..

[57] González-Garrido J.A., García-Sánchez J.R., López-Victorio C.J., Escobar-Ramírez A., Olivares-Corichi I.M. (2023). Cocoa: A Functional Food That Decreases Insulin Resistance and Oxidative Damage in Young Adults with Class II Obesity. Nutr. Res. Pract..

[58] Zulkefli N., Che Zahari C.N.M., Sayuti N.H., Kamarudin A.A., Saad N., Hamezah H.S., Bunawan H., Baharum S.N., Mediani A., Ahmed Q.U. (2023). Flavonoids as Potential Wound-Healing Molecules: Emphasis on Pathways Perspective. Int. J. Mol. Sci..

[59] Nani A., Murtaza B., Khan A.S., Khan N.A., Hichami A. (2021). AOX and Anti-Inflammatory Potential of Polyphenols Contained in Mediterranean Diet in Obesity: Molecular Mechanisms. Molecules.

[60] Taïlé J., Bringart M., Planesse C., Patché J., Rondeau P., Veeren B., Clerc P., Gauvin-Bialecki A., Bourane S., Meilhac O. (2022). AOX Polyphenols of Antirhea Borbonica Medicinal Plant and Caffeic Acid Reduce Cerebrovascular, Inflammatory and Metabolic Disorders Aggravated by High-Fat Diet-Induced Obesity in a Mouse Model of Stroke. AOXs.

[61] Lamia K.A., Sachdeva U.M., DiTacchio L., Williams E.C., Alvarez J.G., Egan D.F., Vasquez D.S., Juguilon H., Panda S., Shaw R.J. (2009). AMPK Regulates the Circadian Clock by Cryptochrome Phosphorylation and Degradation. Science.

[62] Sani M., Sebai H., Ghanem-Boughanmi N., Boughattas N.A., Ben-Attia M. (2015). Circadian (about 24-Hour) Variation in Malondialdehyde Content and Catalase Activity of Mouse Erythrocytes. Redox Rep..

[63] O’Neill J.S., Feeney K.A. (2014). Circadian Redox and Metabolic Oscillations in Mammalian Systems. Antioxid. Redox Signal.

[64] Prasad M.K., Mohandas S., Ramkumar K.M. (2023). Dysfunctions, Molecular Mechanisms, and Therapeutic Strategies of Pancreatic β-Cells in Diabetes. Apoptosis.

[65] Cao J., Yu X., Deng Z., Pan Y., Zhang B., Tsao R., Li H. (2018). Chemical Compositions, Antiobesity, and AOX Effects of Proanthocyanidins from Lotus Seed Epicarp and Lotus Seed Pot. J. Agric. Food Chem..

[66] Fogacci F., Borghi C., Rizzoli E., Giovannini M., Bove M., D’addato S., Cicero A.F.G. (2022). Effect of Dietary Supplementation with Eufortyn^®^ Colesterolo Plus on Serum Lipids, Endothelial Reactivity, Indexes of Non-Alcoholic Fatty Liver Disease and Systemic Inflammation in Healthy Subjects with Polygenic Hypercholesterolemia: The ANEMONE Study. Nutrients.

[67] Sandoval V., Sanz-Lamora H., Arias G., Marrero P.F., Haro D., Relat J. (2020). Metabolic Impact of Flavonoids Consumption in Obesity: From Central to Peripheral. Nutrients.

[68] Ávila-Román J., Soliz-Rueda J.R., Bravo F.I., Aragonès G., Suárez M., Arola-Arnal A., Mulero M., Salvadó M.-J., Arola L., Torres-Fuentes C. (2021). Phenolic Compounds and Biological Rhythms: Who Takes the Lead?. Trends Food Sci. Technol..

[69] Hironao K., Mitsuhashi Y., Huang S., Oike H., Ashida H., Yamashita Y. (2020). Cacao Polyphenols Regulate the Circadian Clock Gene Expression and through Glucagonnlike Peptidee1 Secretion. J. Clin. Biochem. Nutr..

[70] Rodríguez R.M., Cortés-Espinar A.J., Soliz-Rueda J.R., Feillet-Coudray C., Casas F., Colom-Pellicer M., Aragonès G., Avila-Román J., Muguerza B., Mulero M. (2022). Time-of-Day Circadian Modulation of Grape-Seed Procyanidin Extract (GSPE) in Hepatic Mitochondrial Dynamics in Cafeteria-Diet-Induced Obese Rats. Nutrients.

[71] Escobar-Martínez I., Arreaza-Gil V., Muguerza B., Arola-Arnal A., Bravo F.I., Torres-Fuentes C., Suárez M. (2022). Administration Time Significantly Affects Plasma Bioavailability of Grape Seed Proanthocyanidins Extract in Healthy and Obese Fischer 344 Rats. Mol. Nutr. Food Res..

[72] Türk D., Scherer N., Selzer D., Dings C., Hanke N., Dallmann R., Schwab M., Timmins P., Nock V., Lehr T. (2023). Significant Impact of Time-of-Day Variation on Metformin Pharmacokinetics. Diabetologia.

[73] Parkar S.G., Kalsbeek A., Cheeseman J.F. (2019). Potential Role for the Gut Microbiota in Modulating Host Circadian Rhythms and Metabolic Health. Microorganisms.

[74] Branecky K.L., Niswender K.D., Pendergast J.S. (2015). Disruption of Daily Rhythms by High-Fat Diet Is Reversible. PLoS ONE.

[75] Vera L.M., Montoya A., Pujante I.M., Pérez-Sánchez J., Calduch-Giner J.A., Mancera J.M., Moliner J., Sánchez-Vázquez F.J. (2014). Acute Stress Response in Gilthead Sea Bream (*Sparus aurata* L.) Is Time-of-Day Dependent: Physiological and Oxidative Stress Indicators. Chronobiol. Int..

[76] Escribano B.M., Moreno A., Tasset I., Túnez I. (2014). Impact of Light/Dark Cycle Patterns on Oxidative Stress in an Adriamycin-Induced Nephropathy Model in Rats. PLoS ONE.

[77] Man A.W.C., Xia N., Li H. (2020). Circadian Rhythm in Adipose Tissue: Novel AOX Target for Metabolic and Cardiovascular Diseases. AOXs.

[78] Ribas-Latre A., Baselga-Escudero L., Casanova E., Arola-Arnal A., Salvadó M.J., Arola L., Bladé C. (2015). Chronic Consumption of Dietary Proanthocyanidins Modulates Peripheral Clocks in Healthy and Obese Rats. J. Nutr. Biochem..

[79] Ribas-Latre A., Del Bas J.M., Baselga-Escudero L., Casanova E., Arola-Arnal A., Salvadó M.J., Bladé C., Arola L. (2015). Dietary Proanthocyanidins Modulate the Rhythm of BMAL1 Expression and Induce RORα Transactivation in HepG2 Cells. J. Funct. Foods.

[80] Colom-Pellicer M., Rodríguez R.M., Soliz-Rueda J.R., de Assis L.V.M., Navarro-Masip È., Quesada-Vázquez S., Escoté X., Oster H., Mulero M., Aragonès G. (2022). Proanthocyanidins Restore the Metabolic Diurnal Rhythm of Subcutaneous White Adipose Tissue According to Time-Of-Day Consumption. Nutrients.

[81] Hsieh M.C., Yang S.C., Tseng H.L., Hwang L.L., Chen C.T., Shieh K.R. (2010). Abnormal Expressions of Circadian-Clock and Circadian Clock-Controlled Genes in the Livers and Kidneys of Long-Term, High-Fat-Diet-Treated Mice. Int. J. Obes..

[82] Xu Y.Q., Zhang D., Jin T., Cai D.J., Wu Q., Lu Y., Liu J., Klaassen C.D. (2012). Diurnal Variation of Hepatic AOX Gene Expression in Mice. PLoS ONE.

[83] Martin V., María Sainz R., Mayo J.C., Antolín I., Herrera F., Rodríguez C. (2003). Daily Rhythm of Gene Expression in Rat Superoxide Dismutases. Endocr. Res..

[84] Patel S.A., Velingkaar N.S., Kondratov R.V. (2014). Transcriptional Control of AOX Defense by the Circadian Clock. Antioxid. Redox Signal.

[85] Qi G., Wu W., Mi Y., Shi R., Sun K., Li R., Liu X., Liu X. (2018). Tea Polyphenols Direct Bmal1-Driven Ameliorating of the Redox Imbalance and Mitochondrial Dysfunction in Hepatocytes. Food Chem. Toxicol..

[86] McClean C., Davison G.W. (2022). Circadian Clocks, Redox Homeostasis, and Exercise: Time to Connect the Dots?. AOXs.

[87] Wible R.S., Ramanathan C., Sutter C.H., Olesen K.M., Kensler T.W., Liu A.C., Sutter T.R. (2018). NRF2 Regulates Core and Stabilizing Circadian Clock Loops, Coupling Redox and Timekeeping in Mus Musculus. Elife.

[88] Yamakado M., Tanaka T., Nagao K., Imaizumi A., Komatsu M., Daimon T., Miyano H., Tani M., Toda A., Yamamoto H. (2017). Plasma Amino Acid Profile Associated with Fatty Liver Disease and Co-Occurrence of Metabolic Risk Factors. Sci. Rep..

[89] Mardinoglu A., Bjornson E., Zhang C., Klevstig M., Söderlund S., Ståhlman M., Adiels M., Hakkarainen A., Lundbom N., Kilicarslan M. (2017). Personal Model-assisted Identification of NAD + and Glutathione Metabolism as Intervention Target in NAFLD. Mol. Syst. Biol..

[90] Zhou X., Han D., Xu R., Wu H., Qu C., Wang F., Wang X., Zhao Y. (2016). Glycine Protects against High Sucrose and High Fat-Induced Non-Alcoholic Steatohepatitis in Rats. Oncotarget.

[91] Rom O., Liu Y., Liu Z., Zhao Y., Wu J., Ghrayeb A., Villacorta L., Fan Y., Chang L., Wang L. (2020). Glycine-Based Treatment Ameliorates NAFLD by Modulating Fatty Acid Oxidation, Glutathione Synthesis, and the Gut Microbiome. Sci. Transl. Med..

[92] Du K., Hyun J., Premont R.T., Choi S.S., Michelotti G.A., Swiderska-Syn M., Dalton G.D., Thelen E., Rizi B.S., Jung Y. (2018). Hedgehog-YAP Signaling Pathway Regulates Glutaminolysis to Control Activation of Hepatic Stellate Cells. Gastroenterology.

[93] Zheng N., Gu Y., Hong Y., Sheng L., Chen L., Zhang F., Hou J., Zhang W., Zhang Z., Jia W. (2020). Vancomycin Pretreatment Attenuates Acetaminophen-Induced Liver Injury through 2-Hydroxybutyric Acid. J. Pharm. Anal..

[94] Violet P.C., Ebenuwa I.C., Wang Y., Niyyati M., Padayatty S.J., Head B., Wilkins K., Chung S., Thakur V., Ulatowski L. (2020). Vitamin E Sequestration by Liver Fat in Humans. JCI Insight.

[95] Teixeira K.R.C., dos Santos C.P., de Medeiros L.A., Mendes J.A., Cunha T.M., De Angelis K., Penha-Silva N., de Oliveira E.P., Crispim C.A. (2019). Night Workers Have Lower Levels of AOX Defenses and Higher Levels of Oxidative Stress Damage When Compared to Day Workers. Sci. Rep..

[96] Kim T.D., Woo K.C., Cho S., Ha D.C., Sung K.J., Kim K.T. (2007). Rhythmic Control of AANAT Translation by HnRNP Q in Circadian Melatonin Production. Genes. Dev..

[97] Greco C.M., Koronowski K.B., Smith J.G., Shi J., Kunderfranco P., Carriero R., Chen S., Samad M., Welz P.-S., Zinna V.M. (2021). Integration of Feeding Behavior by the Liver Circadian Clock Reveals Network Dependency of Metabolic Rhythms. Sci. Adv..

[98] Ribas-Latre A., Santos R.B., Fekry B., Tamim Y.M., Shivshankar S., Mohamed A.M.T., Baumgartner C., Kwok C., Gebhardt C., Rivera A. (2021). Cellular and Physiological Circadian Mechanisms Drive Diurnal Cell Proliferation and Expansion of White Adipose Tissue. Nat. Commun..

[99] Abe Y.O., Yoshitane H., Kim D.W., Kawakami S., Koebis M., Nakao K., Aiba A., Kim J.K., Fukada Y. (2022). Rhythmic Transcription of Bmal1 Stabilizes the Circadian Timekeeping System in Mammals. Nat. Commun..

