# Supplementary material for: Rhythm and ROS: Hepatic Chronotherapeutic Features of Grape Seed Proanthocyanidin Extract Treatment in Cafeteria Diet-Fed Rats
Source: Antioxidants (Basel). 2023 Aug 12;12(8):1606. doi: 10.3390/antiox12081606 (PMC10452039; doi:10.3390/antiox12081606)
Supplement: Supplementary file 1 [file antioxidants-12-01606-s001.zip › antioxidants-2537641-supplementary.pdf]

Table S1: Weekly food intake of STD- and CAF-fed rats treated at ZT0 or ZT12 with VH or 25 mg/kg b.w. GSPE expressed in g/day and kcal/day.

		**Week 1**	**Week 2**	**Week 3**	**Week 4**	**Week 5**	**Treatment**	**Week 6**	**Week 7**	**Week 8**	**Week 9**
Food intake (g/day)	STD	26.64 ± 1.74	26.13 ± 2.15	25.13 ± 1.74	25.14 ± 2.16	25.08 ± 1.62	ZT0-STD-VH	25.56 ± 1.58	25.53 ± 1.33	25.91 ± 5.25	24.34 ± 1.24
							ZT12-STD-VH	26.26 ± 1.11	25.26 ± 2.09	23.49 ± 1.53	25.15 ± 1.94
	CAF	68.22 ± 9.47^{+++}	64.19 ± 15.61^{+++}	62.76 ± 11.69^{+++}	64.66 ± 17.03^{+++}	57.69 ± 14.55^{+++}	ZT0-CAF-VH	62.85 ± 10.95^{+++}	55.03 ± 7.17^{+++}	57.83 ± 7.96^{+++}	45.19 ± 14.72^{++}
							ZT0-CAF-GSPE	56.84 ± 11.63	47.85 ± 8.24	58.39 ± 12.2	52.68 ± 11.7
							ZT12-CAF-VH	63.09 ± 9.22^{+++}	54.7 ± 13.08^{+++}	58.33 ± 16.33^{+++}	57.14 ± 17.78^{++}
							ZT12-CAF-GSPE	61.54 ± 8.64	53.22 ± 3.4	68.35 ± 15.65	49.95 ± 10.55
Food intake (kcal/day)	STD	82.59 ± 5.41	80.99 ± 6.68	77.9 ± 5.38	77.95 ± 6.71	77.73 ± 5.04	ZT0-STD-VH	79.23 ± 4.9	79.15 ± 4.12	80.31 ± 16.28	70.4 ± 12.83
							ZT12-STD-VH	81.41 ± 3.44	78.31 ± 6.48	72.81 ± 4.76	77.95 ± 6.00
	CAF	332.87 ± 40.79^{+++}	306.64 ± 77.12^{+++}	286.98 ± 55.24^{+++}	302.86 ± 84.24^{+++}	270.07 ± 75.49^{+++}	ZT0-CAF-VH	302.76 ± 78.67^{+++}	248.95 ± 32.08^{+++}	262.8 ± 29.77^{+++}	202.12 ± 67.11^{+++}
							ZT0-CAF-GSPE	278.87 ± 51.05	226.42 ± 31.87	280.49 ± 61.03	246.98 ± 47.69
							ZT12-CAF-VH	296.22 ± 24.37^{+++}	246.08 ± 32.45^{+++}	274.02 ± 76.98^{+++}	256.17 ± 86.1^{+++}
							ZT12-CAF-GSPE	294.41 ± 38.72	249.2 ± 35.14	332.11 ± 83.99	227.1 ± 64.89

Values are expressed as the mean ± SD. (n = 32 and 64 for STD and CAF-fed rats before treatment, respectively; 16 for each experimental group). + indicates significant differences ($p < 0.05$) by diet effect (STD-VH *vs.* CAF-VH) for each ZT treatment; ++ indicates $p < 0.01$; +++ indicates $p < 0.001$. using Student's *t*-test. STD, rats fed a Standard diet; CAF, rats fed a Cafeteria diet; VH, rats administered vehicle; GSPE, rats administered 25 mg/kg b.w. grape seed proanthocyanidin extract.

Table S2: Liver antioxidant-related parameters of STD- and CAF-fed rats treated at ZT0 or ZT12 with VH or 25 mg/kg b.w. GSPE.

			**Sod (U/mg protein)**	**Catalase (U/mg protein)**	**GPx1 (mU/mg protein)**	**GSH (nmol/g fresh tissue)**	**Thiols (nmol SH/mg protein)**
ZT0	ZT1	STD-VH	10.2 (9.6 - 10.56)	711.22 (652.19 - 766.62)	2078.03 (2070.86 - 2084.15)	3133.74 (3018.57 - 3238.67)	119,39 (107,89 - 125,92)
		CAF-VH	11.72 (11.31 - 11.86)	720.95 (710.18 - 745.11)	2105.57 (1844.87 - 2342.46)	1997 (1958.18 - 1998.1) $	86,52 (84,91 - 87,22)
		CAF-GSPE	9.59 (9.4 - 10.07)	628.31 (604.98 - 649.96) #	1346.45 (1275.91 - 1589.75)	2054.13 (1640.3 - 2179.92)	88,17 (82,54 - 104,24)
	ZT7	STD-VH	9.42 (9.3 - 9.63)	661.04 (644.7 - 694.23)	2019.01 (1856.61 - 2234.34)	3038.34 (2824.1 - 3093.92)	104,3 (85,96 - 116,88)
		CAF-VH	10.54 (10.27 - 10.84) +	721.21 (657.94 - 776.14)	1406.69 (1357.37 - 1520.52) +	2274.96 (1542.43 - 3048.87)	108,15 (95,58 - 122,49)
		CAF-GSPE	9.87 (9.73 - 9.96) #	664.39 (642.27 - 716.37)	1619.85 (1372 - 1891.11)	2095.88 (1794.39 - 2439.39)	111,01 (100,52 - 120,92)
	ZT13	STD-VH	13.54 (12.73 - 14.35)	722.29 (714.74 - 741.37)	2494.94 (2360.49 - 2603.32)	2204.43 (2147.76 - 2352.55)	83,76 (80,13 - 87,12)
		CAF-VH	14.76 (14.05 - 15.03)	724.02 (700.86 - 751.46)	2013.04 (1918.87 - 2084.62)	1999.65 (1633.34 - 2344.42)	80,4 (75,58 - 90,36)
		CAF-GSPE	15.84 (14.54 - 17.13)	714.77 (686.64 - 759.4)	2286.9 (2108.09 - 2372.41)	1599.94 (1360.02 - 1797.15)	89,94 (82,09 - 101,64)
	ZT19	STD-VH	13.11 (12.26 - 13.9)	724.14 (695 - 748.1)	2496.15 (2365.07 - 2599.67)	2090.91 (1926.46 - 2198.96)	82,75 (75,51 - 90,19)
		CAF-VH	12.63 (12.62 - 12.73)	683.05 (666.55 - 698.16)	2037.15 (1891.08 - 2191.33)	1862.03 (1633.61 - 2033.14)	84,94 (82,21 - 87,68)
		CAF-GSPE	13.55 (13.06 - 14.21)	650.33 (634.68 - 670.51)	1985.89 (1736.22 - 2334.58)	1494.99 (1348.16 - 1557.59)	79,3 (74,43 - 83,48)
ZT12	ZT1	STD-VH	10.47 (9.67 - 11.64)	721.88 (698.81 - 744.46)	2296.09 (2051.23 - 2441.45)	2758.19 (2320.37 - 3036.7)	109,61 (108,9 - 109,76)
		CAF-VH	10.98 (10.31 - 11.78)	722.19 (717.64 - 726.22)	1686.36 (1617.97 - 1813.18)	2414.3 (2244.1 - 2504.21)	128,37 (116,54 - 139,57) $
		CAF-GSPE	10.3 (10.19 - 10.54)	690.53 (652.68 - 736.07)	1710.86 (1607.78 - 1861.59)	2178.28 (2069.23 - 2416.68)	134,64 (121,78 - 142,2)
	ZT7	STD-VH	10.78 (10.11 - 11.62)	725.53 (694.97 - 742.9)	2296.89 (2125.45 - 2540.07)	3033.77 (2951.82 - 3291.03)	121,5 (114,14 - 133,98)
		CAF-VH	11.13 (10.91 - 11.41)	714.08 (655.4 - 791.33)	1833.63 (1664.38 - 1920.8) +	2365.05 (1974.57 - 2648.96) $	130,15 (123,49 - 134,42)
		CAF-GSPE	10.98 (10.89 - 11.13)	763.3 (741.18 - 773.68)	1843.94 (1698.48 - 1876.91)	2029.77 (1741.65 - 2171.23)	122,96 (114,16 - 131,93)
	ZT13	STD-VH	13.76 (13.43 - 13.89)	662.92 (647.56 - 701.2)	2465.76 (2382.33 - 2553.05)	1812.39 (1800.34 - 1887.77)	91,02 (78,07 - 104,6)
		CAF-VH	12.04 (11.36 - 12.91)	659.39 (644.3 - 674.92)	1700.94 (1512.93 - 1882.4) +	1948.27 (1842.69 - 2090.95)	86,64 (83,22 - 89,49)
		CAF-GSPE	11.16 (10.87 - 11.78)	652.89 (644.57 - 662.34)	1816.09 (1496.1 - 2108.56)	1888.86 (1682.44 - 2074.02)	88,05 (77,49 - 100,42)
	ZT19	STD-VH	13.1 (12.17 - 13.76)	670.24 (651.74 - 690.47)	2344.17 (2107.79 - 2515.45)	2197.5 (1989.25 - 2420.08)	89,77 (84,25 - 94,12)
		CAF-VH	12.98 (12.78 - 13.84)	634.82 (622.26 - 650.85)	1767.47 (1716.32 - 1942.78)	1649.22 (1311.65 - 2059.42)	78,96 (76,32 - 83,3)
		CAF-GSPE	12.79 (12.66 - 12.97)	676.69 (638.55 - 708.27)	1790.95 (1497.62 - 2021.91)	1468.53 (1342.59 - 1606.33)	90,13 (89,53 - 90,84)

Values are expressed as the median (Q1-Q3), (n = 3 - 4) for ZT0 and ZT12 conditions. + indicates significant differences ($p < 0.05$) by diet effect (STD-VH *vs.* CAF-VH), $ indicates tendency ($0.1 > p \geq 0.05$) by diet effect; # tendency ($0.1 > p \geq 0.05$) by treatment effect (CAF-VH *vs.* CAF-GSPE) using Mann-Whitney U test. STD, rats fed a Standard diet; CAF, rats fed a Cafeteria diet; VH, rats administered vehicle; GSPE, rats administered 25 mg/kg b.w. grape seed proanthocyanidin extract.

Table S3: Antioxidant-related metabolites in ZT0-treated rats expressed in arbitrary units.

Metabolite	ZT1			ZT1 p-value	ZT7			ZT7 p-value	ZT13			ZT13 p-value	ZT19			ZT19 p-value	STD-VH p-value	CAF-VH p-value	CAF-GSPE p-value	p-value
	STD-VH	CAF-VH	CAF-GSPE		STD-VH	CAF-VH	CAF-GSPE		STD-VH	CAF-VH	CAF-GSPE		STD-VH	CAF-VH	CAF-GSPE					
2-Hydroxybutyric Acid	0.92 (0.56 - 2.04)	3.98 (3.88 - 4.08)	4.36 (4.26 - 4.38)#	0.140	0.86 (0.81 - 0.97)	1.25 (1.05 - 1.72)$	2.07 (1.62 - 2.57)	0.059	1.23 (0.62 - 2.07)	5.52 (5.27 - 6.67)+	3.98 (3.65 - 4.18)*	0.007	2.39 (2.18 - 2.48)	4.11 (3.93 - 4.2)+	5.02 (4.12 - 5.8)	0.018	0.687	0.005	0.031	0.000
2-Hydroxyisobutyric Acid	1.92 (1.68 - 2.13)	2.5 (2.42 - 2.56)+	2.25 (2.21 - 2.88)	0.076	2.41 (1.74 - 2.94)	2.71 (2.6 - 2.91)	3.34 (3.06 - 3.51)	0.276	2.01 (1.87 - 2.15)	3.25 (3.02 - 3.46)+	2.63 (2.35 - 3.04)	0.049	2.18 (2.08 - 2.32)	2.59 (2.41 - 2.85)$	3.12 (2.91 - 3.36)	0.174	0.867	0.148	0.433	0.034
3-Hydroxybutyric Acid – 3-Hydroxyisobutyric Acid	17 (11.59 - 30.45)	47.06 (37.19 - 58.6)	53.22 (48.36 - 55.43)	0.492	17.09 (14.47 - 20.31)	15.08 (11.73 - 18.66)	29.47 (23.75 - 31.54)	0.841	25.85 (8.87 - 42.88)	47.55 (44.03 - 55.37)$	44.73 (39.42 - 46.77)	0.077	34.98 (34 - 36.27)	34.09 (24.41 - 44.63)	51.45 (42.56 - 66.58)	0.309	0.205	0.041	0.162	0.044
4-Hydroxyphenyllactic Acid	43.6 (42.67 - 44.31)	76.67 (67.36 - 87.66)+	34.19 (24.71 - 58.11)	0.115	38.93 (32.98 - 52.03)	31.91 (23.1 - 43.09)	23.09 (13.19 - 44.11)	0.298	22.36 (16.26 - 29.12)	28.55 (25.55 - 34.27)	30.88 (29.71 - 32.73)	0.368	36.15 (32.84 - 36.95)	33.07 (26.55 - 39.32)	27.1 (24.31 - 34.38)	0.694	0.111	0.059	0.812	0.173
α-Ketoglutaric Acid	5.17 (4.68 - 5.56)	11.82 (8.44 - 15.11)+	6.3 (4.99 - 7.75)	0.899	6.84 (5.87 - 7.39)	3.32 (3.26 - 3.76)+	4.9 (4.07 - 5.54)	0.551	4.84 (4.09 - 5.68)	6.11 (4.87 - 7.94)	6.86 (5.33 - 8.45)	0.368	7.12 (6.63 - 7.42)	8.57 (6.08 - 10.21)	6.39 (5.51 - 7.86)	0.779	0.099	0.350	0.453	0.393
α-Tocopherol	0.05 (0.05 - 0.05)	0.04 (0.04 - 0.04)	0.03 (0.03 - 0.04)	0.268	0.03 (0.02 - 0.03)	0.1 (0.07 - 0.12)+	0.06 (0.05 - 0.06)	0.013	0.05 (0.03 - 0.06)	0.08 (0.07 - 0.09)$	0.04 (0.03 - 0.06)	0.197	0.05 (0.05 - 0.05)	0.06 (0.05 - 0.07)	0.05 (0.04 - 0.05)	0.551	0.162	0.042	0.287	0.017
Citric Acid	77.38 (67.36 - 82.92)	149.78 (128.85 - 183.72)+	70.63 (64.73 - 102.5)#	0.041	75.64 (69.51 - 78.84)	50.44 (45.89 - 52.92)+	60.47 (46.99 - 81.22)	0.472	69.23 (57.71 - 73.45)	61.16 (52.83 - 83.38)	90.54 (79.06 - 99.76)	0.981	104.24 (102.58 - 106.04)	109.89 (95.24 - 115.07)	89.06 (70.94 - 103.78)	0.298	0.034	0.014	0.916	0.030
Dodecanoic Acid	0.27 (0.26 - 0.3)	0.34 (0.28 - 0.38)	0.39 (0.34 - 0.4)	0.260	0.26 (0.24 - 0.28)	0.29 (0.29 - 0.3)	0.3 (0.29 - 0.31)	0.025	0.26 (0.25 - 0.26)	0.3 (0.3 - 0.3)+	0.37 (0.35 - 0.37)*	0.017	0.24 (0.21 - 0.27)	0.33 (0.28 - 0.39)+	0.36 (0.31 - 0.4)	0.033	0.225	0.142	0.222	0.002
Fumaric Acid	2.26 (1.69 - 3.36)	4.49 (4.3 - 4.91)	2.64 (2.24 - 4.17)	0.920	3.01 (2.35 - 3.65)	2.09 (1.73 - 2.32)	2.53 (1.62 - 3.82)	0.874	2.21 (1.67 - 2.79)	2.6 (2.15 - 3.32)	2.84 (2.5 - 3.19)	0.874	2.64 (2.01 - 3.28)	3.12 (2.06 - 3.86)	3.19 (1.87 - 4.43)	0.694	0.750	0.734	0.784	0.979
Glucose-6-phosphate	0.05 (0.04 - 0.06)	0.04 (0.03 - 0.04)	0.03 (0.03 - 0.03)	0.302	0.05 (0.05 - 0.06)	0.04 (0.03 - 0.04)+	0.03 (0.02 - 0.04)	0.089	0.05 (0.05 - 0.05)	0.05 (0.05 - 0.05)	0.04 (0.04 - 0.04)	0.119	0.04 (0.04 - 0.05)	0.03 (0.02 - 0.03)+	0.02 (0.02 - 0.02)	0.017	0.954	0.104	0.261	0.010
Glutamic Acid	9.01 (7.87 - 10.32)	10.17 (9.37 - 10.7)	7.42 (7.38 - 8.87)	0.628	10.84 (9.62 - 11.89)	9.06 (8.36 - 9.2)	9.03 (7.92 - 10.17)	0.276	9.13 (7.66 - 10.7)	10.31 (9.4 - 11.43)	10.24 (8.85 - 11.56)	0.618	10.27 (9.22 - 11.76)	10.68 (9.33 - 11.81)	8.09 (7.52 - 10.06)	0.735	0.657	0.185	0.794	0.740
Glutamine	0.47 (0.43 - 0.51)	0.34 (0.31 - 0.35)+	0.34 (0.34 - 0.39)	0.464	0.38 (0.28 - 0.43)	0.56 (0.48 - 0.66)	0.44 (0.39 - 0.54)	0.351	0.54 (0.44 - 0.73)	0.34 (0.28 - 0.45)$	0.42 (0.38 - 0.51)	0.130	0.39 (0.38 - 0.39)	0.52 (0.44 - 0.59)	0.57 (0.56 - 0.59)	0.064	0.129	0.248	0.260	0.119
Glycine	5.25 (5.16 - 5.28)	5.24 (5.13 - 5.39)	4.89 (4.85 - 5.01)#	0.052	5.21 (4.94 - 5.4)	5.18 (4.81 - 5.54)	4.97 (4.67 - 5.04)	0.703	5.17 (4.96 - 5.29)	4.85 (4.6 - 5.11)	4.99 (4.92 - 5.06)	0.874	4.99 (4.98 - 5.14)	5.07 (4.83 - 5.33)	4.9 (4.57 - 5.28)	0.084	0.688	0.946	0.365	0.402

Metabolite	ZT1			ZT1 p-value	ZT7			ZT7 p-value	ZT13			ZT13 p-value	ZT19			ZT19 p-value	STD-VH p-value	CAF-VH p-value	CAF-GSPE p-value	p-value
	**STD-VH**	**CAF-VH**	**CAF-GSPE**		**STD-VH**	**CAF-VH**	**CAF-GSPE**		**STD-VH**	**CAF-VH**	**CAF-GSPE**		**STD-VH**	**CAF-VH**	**CAF-GSPE**					
Glycolic Acid	0.12 (0.11 - 0.13)	0.15 (0.14 - 0.16)+	0.15 (0.14 - 0.16)	0.313	0.13 (0.12 - 0.14)	0.14 (0.13 - 0.15)	0.13 (0.13 - 0.13)	0.584	0.13 (0.12 - 0.13)	0.14 (0.13 - 0.15)	0.15 (0.14 - 0.17)	0.471	0.11 (0.11 - 0.11)	0.14 (0.14 - 0.15)$	0.14 (0.13 - 0.15)	0.874	0.892	0.897	0.982	0.958
Isoleucine	4.13 (3.98 - 4.18)	3.64 (3.58 - 3.82)	3.58 (3.56 - 3.69)	0.066	3.78 (3.73 - 4.04)	4.41 (4.22 - 4.75)	4.18 (3.84 - 4.52)	0.776	3.73 (3.58 - 3.83)	3.3 (3.02 - 3.7)	3.13 (3.04 - 3.21)	0.078	4 (3.82 - 4.07)	3.27 (3.03 - 3.82)	3.45 (2.83 - 3.98)	0.137	0.491	0.840	0.192	0.056
Lactic acid	75.32 (73.29 - 76.59)	72.95 (68.72 - 79.45)	70.28 (68.8 - 74.97)	0.385	82.06 (79.41 - 83.75)	77.38 (70.97 - 83.28)	70.53 (66.12 - 74.81)	0.167	84.27 (82.09 - 89.04)	95.78 (90.35 - 97.79)	73.19 (67.63 - 78.12)*	0.118	91.09 (87.7 - 95.36)	76.33 (70.18 - 82.36)+	71.22 (64.4 - 81.72)	0.537	0.877	0.014	0.096	0.050
Leucine	6.73 (6.4 - 6.87)	6.13 (5.64 - 6.57)	5.4 (5.34 - 5.78)	0.913	6.08 (5.95 - 6.51)	7.55 (7.03 - 8.03)$	6.62 (6.31 - 7)	0.138	6.43 (6.13 - 6.61)	5.83 (5.22 - 6.49)	5.45 (5.29 - 5.74)	0.059	6.7 (6.23 - 6.86)	5.69 (5.22 - 6.43)	5.45 (4.71 - 6.29)	0.368	0.216	0.138	0.904	0.221
Methionine	3.58 (3.39 - 3.66)	3.27 (3.09 - 3.44)	3.04 (3.02 - 3.08)	0.100	3.37 (3.14 - 3.59)	3.5 (3.37 - 3.57)	3.4 (3.34 - 3.42)	0.500	3.23 (3.18 - 3.31)	2.98 (2.84 - 3.15)	3.07 (2.94 - 3.16)	0.779	3.4 (3.37 - 3.41)	2.97 (2.9 - 3.15)	2.85 (2.44 - 3.3)	0.735	0.623	0.602	0.158	0.412
myo-Inositol	0.92 (0.79 - 1)	0.81 (0.75 - 0.85)	0.64 (0.64 - 0.7)	0.181	0.98 (0.88 - 1.11)	0.84 (0.71 - 0.96)	0.84 (0.71 - 0.93)	0.643	0.92 (0.89 - 0.94)	0.88 (0.86 - 0.89)	0.78 (0.69 - 0.99)	0.537	0.94 (0.92 - 1.03)	0.75 (0.69 - 0.8)+	0.64 (0.62 - 0.71)	0.912	0.400	0.592	0.371	0.505
Ornithine	2.32 (1.96 - 2.96)	1.63 (1.52 - 1.8)$	1.86 (1.78 - 2.06)	0.230	1.88 (1.42 - 2.79)	2.24 (1.86 - 2.86)	1.69 (1.42 - 2.22)	0.432	2.6 (1.96 - 3.15)	1.53 (1.4 - 1.64)+	1.92 (1.72 - 2.07)	0.637	1.33 (1.3 - 1.47)	1.46 (1.14 - 1.88)	1.3 (1.13 - 1.53)	0.025	0.061	0.334	0.538	0.076
Phenylalanine	3.86 (3.6 - 3.88)	3.21 (3 - 3.46)	2.8 (2.79 - 2.98)	0.126	3.59 (3.44 - 3.93)	3.94 (3.78 - 4.08)	3.48 (3.41 - 3.58)*	0.551	3.62 (3.43 - 3.75)	3.35 (3.06 - 3.55)	3.09 (2.91 - 3.27)	0.059	3.75 (3.46 - 4)	2.98 (2.71 - 3.3)	3.07 (2.9 - 3.18)	0.904	0.075	0.172	0.444	0.087
Pyruvic Acid	19.19 (17.3 - 19.73)	21.9 (17.07 - 25.88)	14.13 (14.07 - 17.02)	0.138	25.16 (20.66 - 28.43)	19.73 (17.9 - 23.18)	18.1 (16.35 - 19.38)	0.146	22.38 (20.47 - 24.03)	28.63 (27.13 - 30.29)+	18.59 (15.79 - 22.43)*	0.197	24.11 (24 - 25.02)	21.98 (19.97 - 22.83)+	17.25 (15.62 - 20.41)	0.841	0.136	0.075	0.401	0.024
Serine	9.73 (9.65 - 10.22)	12.1 (11.58 - 13.77)+	11.1 (11 - 11.18)*	0.722	8.61 (8.31 - 9.23)	12.78 (11.04 - 14.74)+	11.89 (11.28 - 12.66)	0.069	9.08 (8.93 - 9.27)	10.52 (9.86 - 11.64)+	12.98 (11.5 - 14.5)	0.926	9.25 (8.86 - 9.62)	11.18 (9.08 - 13.39)	11.01 (10.63 - 12.5)	0.087	0.093	0.991	0.444	0.302
Taurine	0.65 (0.55 - 0.69)	0.49 (0.37 - 0.89)	0.67 (0.49 - 2.38)	0.036	0.75 (0.67 - 0.8)	0.97 (0.76 - 1.21)	0.91 (0.57 - 1.19)	0.037	1.01 (0.94 - 1.22)	0.78 (0.57 - 0.97)$	0.74 (0.68 - 0.83)	0.031	0.84 (0.48 - 1.21)	0.84 (0.74 - 1.08)	0.99 (0.96 - 1.27)	0.118	0.085	0.116	0.229	0.004
Threonine	8.02 (7.61 - 8.39)	8.93 (8.51 - 9.77)	9.04 (8.61 - 9.1)	0.750	7.14 (6.53 - 7.77)	9 (7.97 - 9.75)	8.16 (7.58 - 8.72)	0.298	7.14 (6.8 - 7.56)	6.87 (6.34 - 7.42)	7.77 (7.21 - 8.46)#	0.693	6.72 (6.48 - 6.92)	6.37 (5.57 - 7.69)	6.61 (5.25 - 8.08)	0.472	0.300	0.543	0.304	0.497

Values are expressed as the median (Q1-Q3), (*n = 4*) for ZT0 condition. + indicates significant differences ($p < 0.05$) by diet effect (STD-VH *vs.* CAF-VH), $ indicates tendency ($0.1 > p \geq 0.05$) by diet effect; * indicates significant differences ($p < 0.05$) by treatment effect (CAF-VH *vs.* CAF-GSPE), # indicates tendency

(0.1 > p ≥ 0.05) by treatment effect using Mann-Whitney test. p-value for each group comparison was calculated by Kruskal-Wallis' test. STD, rats fed a Standard diet; CAF, rats fed a Cafeteria diet; VH, rats administered vehicle; GSPE, rats administered 25 mg/kg b.w. grape seed proanthocyanidin extract.

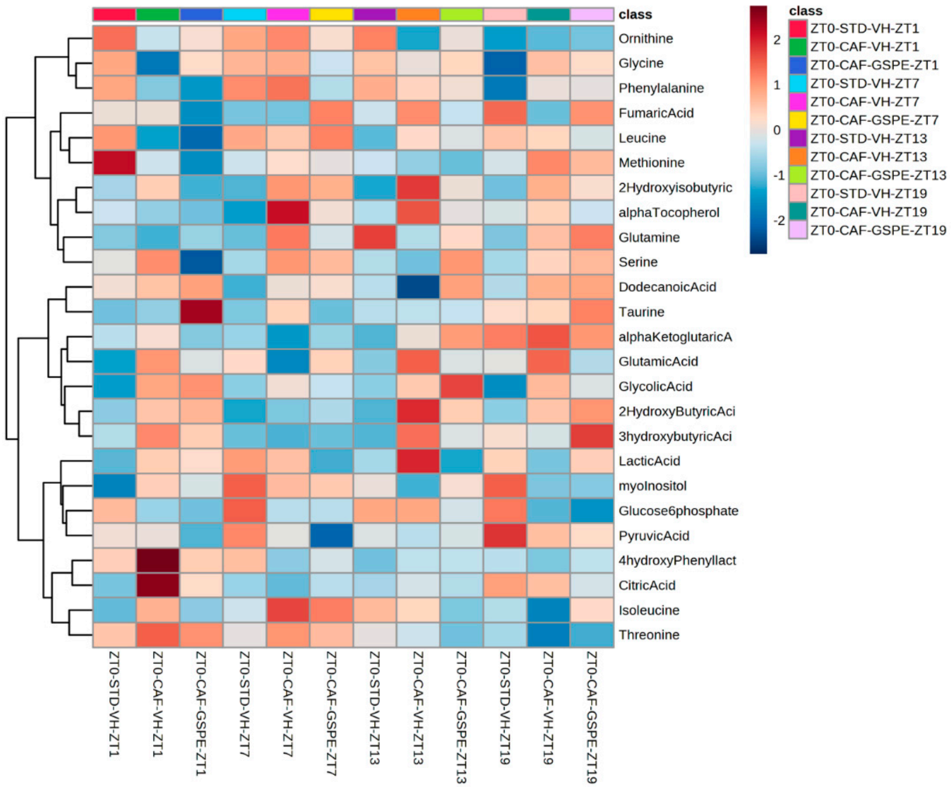

Figure S1: Heatmap analysis of antioxidant-related detected metabolites in liver metabolomics of rats treated at ZT0. STD, rats fed a Standard diet; CAF, rats fed a Cafeteria diet; VH, rats administered vehicle; GSPE, rats administered 25 mg/kg b.w. grape seed proanthocyanidin extract.

Table S4: Antioxidant-related metabolites in ZT12-treated rats expressed in arbitrary units.

Metabolite	ZT1			ZT1	ZT7			ZT7	ZT13			ZT13	ZT19			ZT19				
	STD-VH	CAF-VH	CAF-GSPE	p-value	STD-VH	CAF-VH	CAF-GSPE	p-value	STD-VH	CAF-VH	CAF-GSPE	p-value	STD-VH	CAF-VH	CAF-GSPE	p-value	STD-VH p-value	CAF-VH p-value	CAF-GSPE p-value	p-value
2-Hydroxybutyric Acid	0.63 (0.62 - 0.85)	3.75 (2.76 - 4.28)+	2.16 (1.71 - 2.8)	0.219	0.86 (0.72 - 1.01)	1.38 (1.23 - 1.94)+	1.4 (1.11 - 1.84)	0.083	2.22 (1.81 - 2.67)	3.03 (2.5 - 3.96)	3.26 (2.44 - 4.16)	0.155	3.89 (3.58 - 4.38)	3.84 (3.02 - 4.6)	4.12 (3.8 - 4.65)	0.397	0.361	0.708	0.023	0.046
2-Hydroxyisobutyric Acid	2.14 (2.06 - 2.28)	2.48 (2.01 - 2.83)	2.85 (2.64 - 3.08)	0.174	2.08 (1.78 - 2.3)	2.74 (2.59 - 2.8)+	2.56 (2.4 - 2.72)	0.276	1.94 (1.57 - 2.37)	3.22 (2.95 - 3.31)$	2.95 (2.87 - 3.17)#	0.071	2.09 (2 - 2.6)	2.6 (2.39 - 2.72)	2.6 (2.38 - 2.83)	0.472	0.897	0.162	0.356	0.120
3-Hydroxybutyric Acid – 3-Hydroxyisobutyric Acid	13.65 (10.54 - 19.42)	29.31 (21.95 - 40.08)$	18.95 (16.4 - 21.98)	0.138	12.9 (10.63 - 14.43)	15.74 (13.83 - 21.5)	16.87 (11.86 - 21.42)	0.472	33.3 (27.23 - 41.88)	35.18 (26.06 - 45.44)	43.43 (40.03 - 51.98)	0.219	54.91 (51.46 - 60.4)	41.61 (34.35 - 44.04)+	42.89 (41.85 - 43.31)	0.055	0.009	0.282	0.007	0.001
4-Hydroxyphenyllactic Acid	76.11 (64.13 - 86.98)	48.68 (32.84 - 65.25)	114.34 (82.21 - 144.56)#	0.174	28.43 (26.02 - 40.03)	73.81 (59.57 - 86.39)	50 (41.99 - 51.87)	0.390	33.83 (29.81 - 37.3)	28.89 (22.83 - 41.48)	42.35 (28.82 - 55.48)	0.841	30.38 (24.17 - 36.45)	35.94 (32.4 - 36.68)	41.67 (33.37 - 64.39)	0.472	0.562	0.755	0.027	0.234
α-Ketoglutaric Acid	9.63 (8.99 - 10.52)	7.56 (5.98 - 8.57)$	8.21 (6.82 - 10.31)	0.291	7.38 (6.99 - 7.96)	9.66 (7.36 - 11.33)	5.23 (4.94 - 6.02)	0.199	7.93 (6.21 - 9.88)	5 (4.07 - 7.63)	8.44 (7.64 - 8.81)	0.874	5.87 (4.9 - 6.81)	6.61 (5.74 - 7.51)	9.7 (8.01 - 11.27)	0.584	0.108	0.589	0.792	0.613
α-Tocopherol	0.05 (0.03 - 0.08)	0.06 (0.05 - 0.08)	0.06 (0.06 - 0.06)	0.263	0.04 (0.04 - 0.04)	0.07 (0.06 - 0.08)+	0.09 (0.05 - 0.12)	0.081	0.05 (0.04 - 0.05)	0.08 (0.06 - 0.08)	0.07 (0.05 - 0.09)	0.333	0.05 (0.05 - 0.05)	0.07 (0.07 - 0.08)+	0.09 (0.08 - 0.09)	0.025	0.490	0.898	0.530	0.059
Citric Acid	130.5 (109.15 - 138.59)	80.42 (73.26 - 97.38)	123.05 (112.44 - 143.68)	0.390	95.61 (81.28 - 118.58)	111.99 (102.49 - 118.32)	67.62 (61.67 - 90.98)	0.298	109.67 (93.88 - 131.46)	71.54 (57.9 - 101.71)	125.84 (110.52 - 132.61)	0.116	93.52 (87.92 - 94.81)	96.16 (87.22 - 102.91)	132.93 (115.14 - 159.09)	0.232	0.642	0.080	0.290	0.196
Dodecanoic Acid	0.07 (0.07 - 0.08)	0.16 (0.14 - 0.17)+	0.2 (0.18 - 0.25)	0.014	0.08 (0.07 - 0.08)	0.14 (0.11 - 0.17)	0.2 (0.16 - 0.26)	0.053	0.11 (0.1 - 0.12)	0.19 (0.16 - 0.25)+	0.21 (0.17 - 0.23)*	0.083	0.1 (0.09 - 0.1)	0.23 (0.19 - 0.25)+	0.19 (0.16 - 0.24)	0.024	0.969	0.189	0.599	0.002
Fumaric Acid	3.56 (3.2 - 4.48)	2.93 (2.56 - 3.89)	3.62 (2.52 - 4.75)	0.442	3.74 (2.79 - 4.77)	3.17 (2.49 - 4.45)	2.59 (2.24 - 3.05)	0.437	2.51 (2.38 - 2.63)	2.1 (1.58 - 2.87)	2.63 (2.15 - 3.05)	0.926	2.27 (1.81 - 2.66)	2.38 (2.02 - 2.66)	3.08 (2.85 - 4.03)#	0.103	0.298	0.718	0.423	0.578
Glucose-6-phosphate	0.1 (0.08 - 0.1)	0.04 (0.03 - 0.05)$	0.04 (0.03 - 0.05)	0.858	0.06 (0.06 - 0.06)	0.06 (0.05 - 0.06)	0.05 (0.04 - 0.05)	0.193	0.06 (0.05 - 0.08)	0.03 (0.02 - 0.03)+	0.05 (0.04 - 0.06)*	0.020	0.04 (0.03 - 0.04)	0.02 (0.02 - 0.02)$	0.03 (0.02 - 0.04)	0.318	0.084	0.015	0.170	0.009
Glutamic Acid	11.17 (10.2 - 11.46)	9.16 (7.96 - 10.33)$	8.69 (7.68 - 9.75)	0.668	10.12 (9.42 - 11.02)	9.22 (9.05 - 9.39)	10.16 (9.69 - 10.27)	0.319	10.24 (9.13 - 11.65)	8.54 (7.72 - 9.67)	9.65 (9.05 - 11.16)	0.390	9.66 (9.11 - 10.29)	9.87 (9.49 - 10.33)	10.78 (9.92 - 11.61)	0.146	0.165	0.657	0.159	0.267

Metabolite	ZT1			ZT1	ZT7			ZT7	ZT13			ZT13	ZT19			ZT19	STD-VH	CAF-VH	CAF-GSPE	p-value
	STD-VH	CAF-VH	CAF-GSPE	p-value	STD-VH	CAF-VH	CAF-GSPE	p-value	STD-VH	CAF-VH	CAF-GSPE	p-value	STD-VH	CAF-VH	CAF-GSPE	p-value	p-value	p-value	p-value	
Glutamine	0.45 (0.4 - 0.5)	0.52 (0.38 - 0.8)	0.44 (0.32 - 0.58)	0.861	0.77 (0.69 - 0.83)	0.47 (0.45 - 0.56)	0.57 (0.51 - 0.64)	0.123	0.37 (0.35 - 0.46)	0.8 (0.64 - 1)+	0.51 (0.45 - 0.55)*	0.397	0.51 (0.45 - 0.59)	0.67 (0.59 - 0.75)$	0.67 (0.47 - 0.97)	0.608	0.046	0.967	0.616	0.623
Glycine	5.55 (5.47 - 5.63)	5.12 (4.83 - 5.34)+	4.8 (4.44 - 5.09)	0.491	5.26 (5.02 - 5.34)	4.5 (4.34 - 4.84)	5.32 (4.95 - 5.46)	0.794	5.2 (5.05 - 5.37)	4.75 (4.62 - 4.79)+	5.15 (5.13 - 5.22)*	0.551	4.87 (4.82 - 4.96)	5.14 (4.85 - 5.27)	4.85 (4.8 - 4.98)	0.393	0.159	0.332	0.703	0.413
Glycolic Acid	0.08 (0.07 - 0.09)	0.11 (0.1 - 0.12)+	0.11 (0.11 - 0.12)	0.039	0.1 (0.09 - 0.1)	0.13 (0.11 - 0.14)+	0.12 (0.11 - 0.12)	0.694	0.08 (0.08 - 0.09)	0.12 (0.12 - 0.12)+	0.11 (0.11 - 0.11)*	0.024	0.09 (0.08 - 0.09)	0.12 (0.11 - 0.12)+	0.11 (0.1 - 0.13)	0.904	0.045	0.985	0.595	0.121
Isoleucine	4.79 (4.45 - 5.07)	4.41 (4.19 - 4.72)	4.42 (4.32 - 4.6)	0.141	4.73 (4.19 - 5.05)	4.37 (4.01 - 4.81)	4.1 (3.87 - 4.47)	0.109	3.46 (3.13 - 3.74)	3.67 (3.03 - 4.3)	3.39 (3.28 - 3.46)	0.017	3.18 (2.8 - 3.58)	3.5 (3.4 - 3.6)	3.63 (3.33 - 3.9)	0.594	0.770	0.458	0.979	0.071
Lactic acid	76.32 (70.53 - 82.04)	75.08 (72.98 - 80.84)	87.36 (81.85 - 94.69)	0.511	95.72 (87.36 - 101.69)	104.51 (96.9 - 107.52)	84.8 (78.29 - 94.92)	0.874	80.02 (79.36 - 81.49)	93.95 (83.03 - 106.22)	82.76 (81.61 - 85.72)	0.491	88.69 (82.2 - 95.09)	88.45 (84.42 - 95.42)	94.82 (90.46 - 99.66)	0.944	0.016	0.037	0.094	0.003
Leucine	7.73 (6.98 - 8.5)	7.02 (6.67 - 7.52)	7.27 (6.94 - 7.54)	0.926	7.46 (6.95 - 7.75)	7.22 (6.7 - 7.75)	6.79 (6.18 - 7.5)	0.551	5.71 (5.24 - 6.17)	6.21 (5.35 - 6.94)	5.58 (5.41 - 5.74)	0.841	5.22 (4.82 - 5.65)	5.45 (5.39 - 5.58)	6.17 (5.97 - 6.33)*	0.944	0.205	0.122	0.819	0.397
Methionine	3.72 (3.61 - 3.84)	3.36 (3.25 - 3.48)+	3.22 (3.13 - 3.32)	0.841	3.31 (3.17 - 3.51)	3.28 (3.24 - 3.38)	3.24 (3.15 - 3.39)	0.794	2.97 (2.91 - 3.11)	3.07 (2.94 - 3.23)	2.95 (2.86 - 3.01)	0.437	3.01 (2.98 - 3.03)	3.03 (2.93 - 3.08)	2.85 (2.76 - 3)	0.058	0.168	0.053	0.033	0.015
myo-Inositol	1.16 (1 - 1.33)	0.84 (0.77 - 0.9)+	0.79 (0.75 - 0.82)	0.025	0.91 (0.79 - 1.13)	0.91 (0.84 - 0.97)	0.95 (0.89 - 0.97)	0.828	0.9 (0.84 - 0.99)	0.7 (0.62 - 0.76)+	0.74 (0.68 - 0.82)*	0.939	0.69 (0.63 - 0.74)	0.76 (0.73 - 0.77)$	0.77 (0.72 - 0.81)	0.779	0.006	0.190	0.094	0.006
Ornithine	6.18 (5.14 - 6.99)	3.07 (2.85 - 3.39)+	3.04 (2.71 - 3.32)	0.024	2.85 (2.49 - 3.96)	3.22 (3 - 3.47)	2.47 (2.42 - 2.6)*	0.967	2.84 (2.59 - 3.04)	2.5 (1.94 - 3.08)	2.49 (2.38 - 2.52)	0.023	1.97 (1.8 - 2.16)	2.08 (1.86 - 2.5)	2.09 (1.98 - 2.19)	0.170	0.048	0.187	0.170	0.016
Phenylalanine	3.86 (3.83 - 3.95)	3.52 (3.43 - 3.63)+	3.22 (3.12 - 3.31)*	0.064	3.92 (3.64 - 4.16)	3.76 (3.67 - 3.82)	3.51 (3.23 - 3.78)	0.138	3.28 (3.09 - 3.48)	3.33 (2.94 - 3.66)	2.95 (2.84 - 3.04)	0.492	3.09 (2.98 - 3.13)	2.78 (2.77 - 2.87)	3.18 (2.99 - 3.25)	0.668	0.024	0.195	0.378	0.018
Pyruvic Acid	15.79 (14.32 - 17.86)	16.75 (13.77 - 19.06)	16.98 (14.1 - 20.44)	0.024	21.54 (20.17 - 22.63)	25.62 (20.68 - 29.33)	19.53 (18.39 - 20.35)	0.123	15.41 (15.08 - 15.72)	20.98 (18.59 - 22.72)	15.25 (14.9 - 16.7)	0.292	18.96 (13.97 - 23.81)	18.97 (17.98 - 20.2)	17.2 (14.4 - 19.85)	0.127	0.023	0.463	0.306	0.032
Serine	10.75 (10.39 - 11.26)	12.04 (11.86 - 12.42)+	13.27 (12.49 - 13.92)	0.735	8.71 (8.25 - 9.28)	12.8 (10.91 - 14.64)+	14.17 (11.69 - 16.53)	0.437	8.75 (8.43 - 9.07)	10.41 (9.66 - 11.2)+	10.34 (10.06 - 10.84)*	0.584	8.04 (7.73 - 8.54)	10.66 (10.59 - 11.59)+	11.33 (10.44 - 12.54)	0.491	0.150	0.178	0.523	0.295
Taurine	0.72 (0.3 - 1.54)	1.23 (0.89 - 1.49)	0.41 (0.28 - 0.58)	0.031	1.45 (1.37 - 1.51)	0.72 (0.56 - 1.17)	0.99 (0.83 - 1.36)	0.050	0.64 (0.58 - 0.78)	0.99 (0.94 - 1.08)	0.95 (0.8 - 1.46)	0.105	0.79 (0.64 - 1.04)	0.87 (0.62 - 1.07)	0.84 (0.5 - 1.18)	0.472	0.267	0.117	0.042	0.005
Threonine	9 (8.5 - 9.31)	8.85 (8.48 - 9.63)	7.69 (7.21 - 8.18)	0.694	7.39 (6.72 - 8.23)	8.66 (8.11 - 9.72)+	9.18 (8.43 - 10.46)	0.334	7.02 (6.1 - 7.63)	6.97 (6.45 - 7.56)	6.69 (6.17 - 7.51)	0.481	5.46 (5.23 - 5.8)	7.18 (6.66 - 7.67)+	7.04 (5.97 - 8.12)	0.779	0.086	0.954	0.368	0.556

Values are expressed as the median (Q1-Q3), ($n = 4$) for ZT0 condition. + indicates significant differences ($p < 0.05$) by diet effect (STD-VH *vs.* CAF-VH), $ indicates tendency ($0.1 > p \geq 0.05$) by diet effect; * indicates significant differences ($p < 0.05$) by treatment effect (CAF-VH *vs.* CAF-GSPE), # indicates tendency ($0.1 > p \geq 0.05$) by treatment effect using Mann-Whitney test. p-value for each group comparison was calculated by Kruskal-Wallis' test. STD, rats fed a Standard diet; CAF, rats fed a Cafeteria diet; VH, rats administered vehicle; GSPE, rats administered 25 mg/kg b.w. grape seed proanthocyanidin extract.

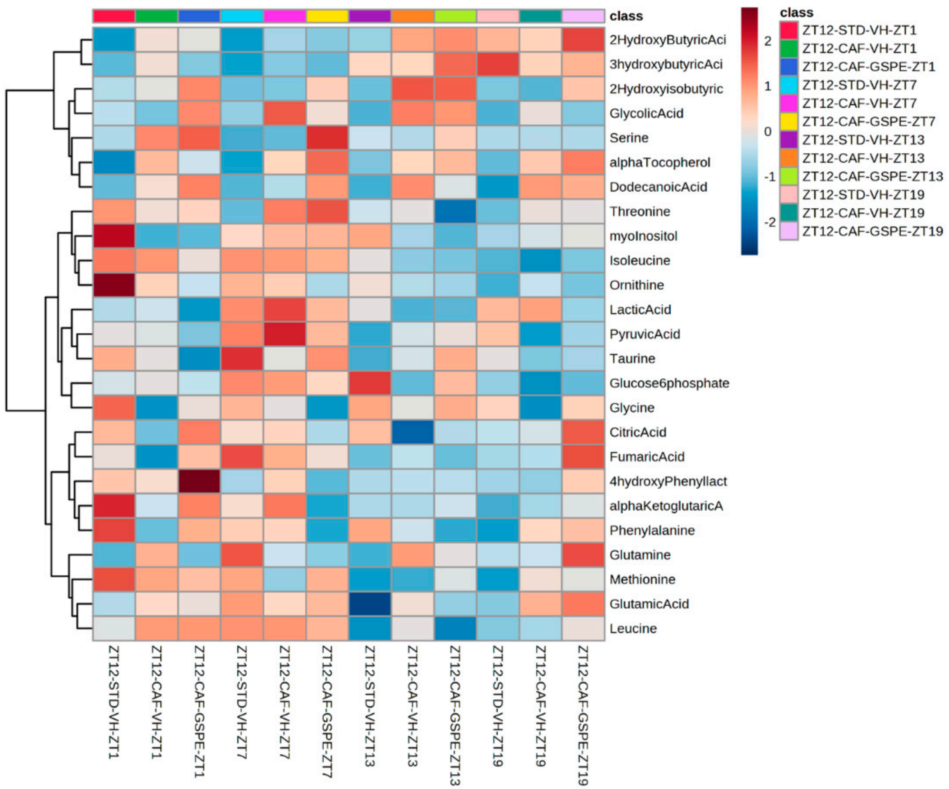

Figure S2: Heatmap analysis of antioxidant-related detected metabolites in liver metabolomics of rats treated at ZT0. STD, rats fed a Standard diet; CAF, rats fed a Cafeteria diet; VH, rats administered vehicle; GSPE, rats administered 25 mg/kg b.w. grape seed proanthocyanidin extract.
